# Chitosan–Silica Composites: Effect of Polymer Loading and Immobilization Strategy on Surface Structure and Ni(II)/Co(II) Adsorption

**DOI:** 10.3390/polym18212610

**Published:** 2026-10-22

**Authors:** Saniya Saryuisin, Eldar Talgatov, Assemgul Auyezkhanova, Aigul Zamanbekova, Zhanar Korganbayeva, Erik Sailaubay

**Affiliations:** 1 Dmitry Vladimirovich Sokolsky Institute of Fuel, Catalysis, and Electrochemistry, Kunaev Str. 142, Almaty 050010, Kazakhstan; 2 Department of Chemistry, Faculty of Natural Science and Geography, Abai Kazakh National Pedagogical University, Kazybek Bi Str. 30, Almaty 050010, Kazakhstan

**Keywords:** chitosan–SiO_2_ composites, chitosan loading, surface modification, APTES functionalization, Ni(II) and Co(II) adsorption, polymer immobilization

## Abstract

In this work, the effects of chitosan loading and immobilization strategy on the structure and Ni(II) and Co(II) adsorption properties of chitosan–silica composites were studied. To examine the effect of polymer loading, CS/SiO_2_ composites containing 5–20 wt.% chitosan were prepared by pH-induced precipitation of chitosan onto pristine SiO_2_. The effect of the immobilization strategy was investigated using an alternative approach involving preliminary functionalization of SiO_2_ with APTES followed by chitosan deposition. The obtained materials were characterized by TGA, elemental analysis, FTIR spectroscopy, nitrogen adsorption–desorption analysis, SEM, and XPS. Direct precipitation of chitosan onto SiO_2_ resulted in the formation of smaller polymer-containing domains on the silica surface compared with the large agglomerates of pristine chitosan, while regions retaining the characteristic morphology of the silica support remained visible. Chitosan modification improved metal-ion adsorption already at a loading of 5 wt.%. Further increasing the chitosan content to 20 wt.% produced a comparatively smaller additional effect. Preliminary APTES functionalization promoted a more uniform surface morphology of CS/SiO_2_–APTES compared with directly prepared CS/SiO_2_. These morphological differences were accompanied by differences in the spectral response of the composites following metal adsorption. CS/SiO_2_–APTES exhibited smaller shifts in the main N 1s XPS component and almost no change in the silanol-related FTIR band, in contrast to the more pronounced changes observed for CS/SiO_2_. The directly prepared composite also exhibited faster initial metal uptake. Nevertheless, both composites showed relatively similar equilibrium metal uptake values. These findings highlight the importance of considering polymer organization and surface chemistry when designing supported polymer composites for adsorption and other potential applications.

## 1. Introduction

Nickel and cobalt are strategically important metals widely used in metallurgy, electrochemistry, catalysis, energy-storage technologies, and the production of high-performance alloys [1,2,3,4,5]. The continuing development of battery and renewable-energy systems is driving further growth in their consumption. According to recent forecasts, global nickel demand may reach approximately 10.3 million tonnes by 2040, while cobalt demand for lithium-ion batteries is projected to exceed 300 thousand tonnes by 2030 [6,7,8]. The increasing production, processing, and use of these metals are accompanied by the generation of wastewater containing dissolved Ni(II) and Co(II) ions, particularly in mining, metallurgy, electroplating, battery manufacturing, and waste-processing industries [9,10,11]. Consequently, the development of effective technologies for the removal and potential recovery of nickel and cobalt from aqueous media remains an important environmental and technological challenge.

Various physicochemical methods, including chemical precipitation, membrane filtration, ion exchange, electrochemical treatment, solvent extraction, coagulation–flocculation, and adsorption, have been employed for the treatment of metal-containing wastewater [12,13,14]. Among these approaches, adsorption is attractive because of its operational simplicity, efficiency, broad applicability, and suitability for both metal removal and recovery [15,16]. In this context, biopolymer-based materials have received considerable attention because they combine renewable origin and environmental compatibility with the presence of functional groups capable of binding metal ions [16,17].

Chitosan, a natural polysaccharide obtained by the deacetylation of chitin, is considered a promising functional component of such materials. Its amino and hydroxyl groups can participate in coordination, electrostatic interactions, and hydrogen bonding with metal ions [18,19]. However, the direct application of pristine chitosan is restricted by its relatively low mechanical stability, swelling, solubility under acidic conditions, and low specific surface area [19,20]. Numerous modification strategies have therefore been developed to improve the physicochemical and functional properties of chitosan-based materials [20,21,22].

Two general approaches have been widely explored to overcome the limitations of pristine chitosan: chemical modification of the polymer itself and its immobilization on inorganic or porous supports.

Considerable efforts have also been devoted to controlling the properties of chitosan-based materials through chemical modification of the polymer. The principal approaches include grafting, crosslinking, and the introduction of additional metal-binding functionalities. Formation of Schiff-base derivatives is commonly used to modify the chemical environment of amino groups and introduce new coordination sites. Crosslinking, particularly with glutaraldehyde, is generally applied to improve the chemical and mechanical stability of the polymer, although it may also alter the availability of its original amino groups [22,23]. For example, Abdelwahab et al. [24] prepared quinoxaline Schiff-base derivatives of chitosan and subsequently crosslinked the modified polymer with glutaraldehyde. Nematidil et al. [25] combined glutaraldehyde-crosslinked chitosan with APTES-modified montmorillonite nanoparticles, producing a porous Schiff-base composite containing several types of functional sites. Such approaches are primarily intended to change the chemical nature, number, or affinity of coordination groups and to stabilize the polymer structure.

Immobilization of chitosan on silica-based supports represents a complementary strategy for improving the structural and surface properties of the resulting materials. Silica is particularly suitable for this purpose because of its developed surface area, tunable pore structure, chemical stability, and the presence of surface silanol groups capable of interacting with the functional groups of chitosan. Several preparation routes have been reported for chitosan–silica materials. Budnyak et al. [26] synthesized a chitosan–silica nanocomposite by the sol–gel method through the in situ formation of an inorganic network from tetraethoxysilane in the presence of a preformed chitosan phase. Błachnio and Zienkiewicz-Strzałka [27] immobilized chitosan on mesoporous cellular silica foams prepared under different sol–gel and ageing conditions, demonstrating the role of the silica scaffold in determining the structural, morphological, and surface properties of the resulting hydrogels. Preliminary functionalization of silica with organosilanes provides another means of controlling the organic–inorganic interface. In particular, Ramasamy et al. [28] employed APTES-functionalized silica in the preparation of chitosan-containing hybrid adsorbents and evaluated the effects of the silane, ligand, and chitosan characteristics on the properties of the resulting materials. Singhon et al. [29] developed silica–chitosan composites for the removal of Ni(II) ions from aqueous solutions. One material was prepared by coating chitosan onto acid-treated silica, while the second was obtained by modifying the silica surface with triethoxysilylbutyronitrile, followed by hydrolysis of the nitrile groups to carboxyl groups and subsequent grafting of chitosan. The obtained composites showed promise as sorbents for Ni(II) removal.

Taken together, these studies demonstrate that chitosan-containing inorganic materials can be prepared through several conceptually different approaches, including chemical modification and crosslinking of the polymer, in situ sol–gel hybridization, immobilization on preformed porous supports, and hybridization involving organosilane-functionalized surfaces. While these approaches provide considerable control over the composition of chitosan-based materials, their functional properties may also depend on the morphology and organization of the polymer-containing phase and how its functional groups are presented at the solid–solution interface. However, substantial differences in the composition and preparation conditions of the reported systems make it difficult to distinguish the individual effects of polymer loading and support prefunctionalization. In particular, the relationship between chitosan loading, the morphology of the polymer-containing phase, and the resulting textural properties of supported chitosan materials remains insufficiently understood. Moreover, direct comparisons of chitosan immobilization on pristine and APTES-functionalized silica under otherwise comparable preparation conditions are scarce. Consequently, it remains unclear whether increasing the amount of immobilized chitosan leads to a proportional improvement in metal-ion adsorption performance, and whether changes in the organization of the polymer phase induced by different immobilization strategies translate into improved adsorption performance. Addressing these questions requires comparing the effects of polymer loading and immobilization strategy under comparable preparation and adsorption conditions. Metal-ion adsorption can therefore serve as a functional probe of how changes in polymer loading and immobilization strategy affect the properties of the resulting materials.

In this work, the effects of chitosan loading and immobilization strategy on the adsorption of Ni(II) and Co(II) by the resulting composites were comparatively evaluated. The effect of polymer loading was examined using a series of CS/SiO_2_ composites with different chitosan contents. The effect of the immobilization strategy was evaluated by comparing composites with similar polymer contents prepared by depositing chitosan directly onto pristine silica (CS/SiO_2_) or onto silica prefunctionalized with APTES (CS/SiO_2_–APTES). The observed differences in metal-ion adsorption were interpreted based on the results of TGA, FTIR spectroscopy, nitrogen adsorption–desorption analysis, SEM, and XPS, allowing them to be related to the content and distribution of chitosan, the textural characteristics of the composites, and the chemical state of the surface functional groups.

## 2. Materials and Methods

### 2.1. Materials

Chitosan (CS, from shrimp shells, 83% deacetylated, Sigma-Aldrich, St. Louis, MO, USA), silica mesostructured (SiO_2_, high-purity grade, 200–400 mesh, Sigma-Aldrich, St. Louis, MO, USA), (3-aminopropyl)triethoxysilane (APTES, 99%, Sigma-Aldrich, St. Louis, MO, USA), hydrochloric acid (HCl, 37%, pure grade, Sigma-Aldrich, St. Louis, MO, USA), sodium hydroxide (NaOH, pure grade, Sigma-Aldrich, St. Louis, MO, USA), and ethanol (96.3%, pure grade, Talgar Alcohol LLP, Talgar, Kazakhstan) were used for preparation of composite sorbents. Nickel (II) chloride hexahydrate (NiCl_2_·6H_2_O, 99.9%, Sigma-Aldrich, St. Louis, MO, USA) and cobalt (II) chloride hexahydrate (CoCl_2_·6H_2_O, 99%, Guangzhou Jinhuada Chemical Reagent Co. Ltd., Guangzhou, China) were used for the adsorption experiments. Dimethylglyoxime (analytical grade, Sigma-Aldrich, St. Louis, MO, USA), sodium thiosulfate (99%, Sigma-Aldrich, St. Louis, MO, USA), and potassium sodium tartrate tetrahydrate (99%, Sisco Research Laboratories Pvt. Ltd., Mumbai, India) were used for the spectrophotometric determination of Ni(II) concentrations in aqueous solutions. Sodium diethyldithiocarbamate (DDTC, analytical grade, Sigma-Aldrich, St. Louis, MO, USA) and aqueous ammonia (25%, Sigma-Aldrich, St. Louis, MO, USA) were used for the spectrophotometric determination of Co(II) concentrations.

### 2.2. Preparation of CS/SiO_2_ Composites

CS/SiO_2_ composites were prepared by a precipitation method as follows. A 1 wt.% chitosan solution was prepared by dissolving 1 g of chitosan in 100 mL of 1% HCl. An appropriate volume (5–25 mL) of the chitosan solution was added dropwise to a well-dispersed suspension of SiO_2_ (1.0 g in 15–35 mL of distilled water) under continuous stirring. After addition of the chitosan solution, the suspension was stirred for 1 h. Under continuous stirring, the pH was adjusted to 7.2–7.6 by the dropwise addition of a 12.5 M NaOH solution, and the suspension was then stirred for an additional 2 h. The stirring was then stopped, and the resulting flocculent precipitate was allowed to settle. The clear supernatant was collected for determination of the residual chitosan concentration. The solid phase was subsequently separated by filtration, thoroughly washed with distilled water, and dried at room temperature to constant mass. The dried composites were ground to a homogeneous powder using a PULVERISETTE 0 vibratory micro mill (Fritsch GmbH, Idar-Oberstein, Germany).

**Figure 1:**
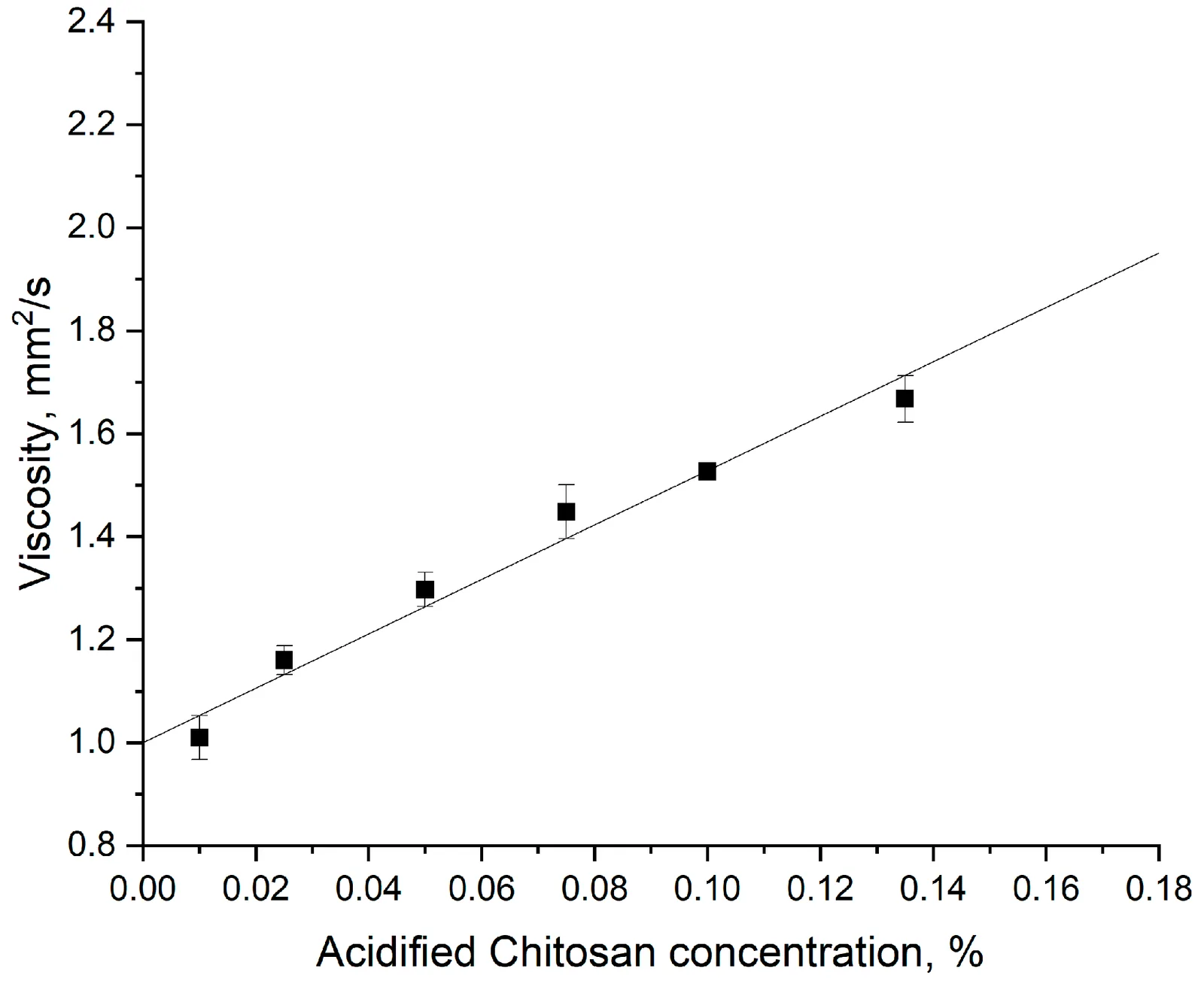
Calibration curve for the determination of residual chitosan concentration by kinematic viscosity measurements.

The chitosan content in the composites was estimated from the difference between the initial amount introduced into the system and that remaining in the supernatant. The residual chitosan concentration in the supernatant was determined by viscometry using a calibration curve describing the dependence of kinematic viscosity on chitosan concentration (Figure 1). Viscosity measurements were carried out at 20 °C using an Ubbelohde viscometer (the manufacturer-specified instrument error was ±0.2%) and a thermostatic bath (KRIO-VIS-E-01, Termex, Tomsk, Russia). The flow time was measured at least three times for each sample, and the average value was used to calculate the viscosity.

To evaluate the possible effect of salts formed during neutralization, blank solutions containing amounts of HCl corresponding to those introduced with the chitosan solutions during preparation of the 5, 10, and 20 wt.% CS/SiO_2_ composites were prepared without chitosan and neutralized to approximately pH 7.5. Their kinematic viscosities were 1.001–1.002 mm^2^/s, i.e., close to that of water under the same measurement conditions, indicating that the salt matrix had only a minor effect on the measured viscosity. The viscometric method was used as a semiquantitative estimate of the residual chitosan concentration.

### 2.3. Preparation of SiO_2_-APTES and CS/SiO_2_-APTES Composites

SiO_2_ was functionalized with APTES by a silanization method in an ethanol medium according to a previously reported procedure [30] with slight modifications. Briefly, 2.7 mL of APTES was added dropwise to a suspension of SiO_2_ (1.0 g) in ethanol (30 mL) under continuous stirring. The suspension was stirred for 3 h at room temperature, after which the pH was adjusted to 8.5–9.0 using 12.5 M NaOH solution. The suspension was then aged at room temperature for 12 h. The resulting SiO_2_-APTES was separated by filtration, washed three times with ethanol, dried at room temperature, and heat-treated at 110 °C for 30 min in a LGYT4-13 muffle furnace (Cangzhou Lovely Technology Co. Ltd., Cangzhou, China). The obtained SiO_2_–APTES was subsequently used for the preparation of the CS/SiO_2_–APTES composite following the same precipitation procedure as described above for the CS/SiO_2_ composites. Briefly, 11 mL of a 1 wt.% chitosan solution was added to a suspension containing 1.0 g of SiO_2_–APTES, followed by pH adjustment to 7.5 and further treatment according to the procedure described above. For comparison, a control sample denoted SiO_2_–APTES-treated was prepared by subjecting SiO_2_–APTES to the same treatment used for chitosan deposition, except that 11 mL of a 1 wt.% HCl solution was added instead of the chitosan solution. The pH was then adjusted to 7.5, and the subsequent treatment steps were performed as described above.

### 2.4. Characterization of the Composites by Physicochemical Methods

Thermogravimetric analysis (TGA) of the composites was carried out using an STA 449 F5 analyzer (NETZSCH, Selb, Germany) under nitrogen flow (50 mL/min) from 30 to 900 °C at a heating rate of 20 °C/min.

The elemental composition of the samples was determined by organic elemental analysis using a Vario Micro Cube elemental analyzer (Elementar Analysensysteme GmbH, Langen-Selbold, Germany) at 1150 °C under a stream of pure oxygen. The combustion products were subsequently reduced and separated using a chromatographic column equipped with a thermal conductivity detector.

Fourier Transform Infrared (FTIR) spectra were obtained using a Nicolet iS5 from Thermo Scientific (Waltham, MA, USA), with a resolution of 3 cm^−1^ in the 4000–400 cm^−1^ region. Pellets for infrared analysis were obtained by grinding a mixture of 1 mg sample with 100 mg dry KBr, followed by pressing the mixture into a mold.

The specific surface area and pore characteristics of the samples were investigated by nitrogen adsorption–desorption at 77 K using a SURFER gas adsorption analyzer (Thermo Fisher Scientific S.p.A., Rodano, Italy). Before analysis, the samples were degassed at 80 °C for 300 min. The BET fitting range was selected individually for each sample in the low-relative-pressure region, approximately (P/P_0_ = 0.05–0.30), based on the Rouquerol consistency criteria and the quality of the linear fit. Specifically, n(1–P/P_0_) was required to increase continuously with increasing relative pressure, the BET constant *C* had to be positive, and the relative pressure corresponding to the calculated monolayer capacity had to lie within the selected fitting range. The total pore volume was estimated by the Gurvich method from the amount of nitrogen adsorbed at P/P_0_ = 0.95. The pore-size distributions were calculated by the Barrett–Joyner–Halenda method from the desorption branch over P/P_0_ = 0.30–0.95 using the universal Halsey thickness equation.

Scanning electron microscopy (SEM) micrographs were obtained on a scanning electron microscope JSM-6610 LV (“JEOL” Ltd., Tokyo, Japan) at an accelerating voltage of 15–20 kV.

X-ray photoelectron spectroscopy (XPS) measurements were performed using an ESCALAB 250Xi X-ray and ultraviolet photoelectron spectrometer (Thermo Fisher Scientific, Waltham, MA, USA) with Al Kα radiation (photon energy, 1486.6 eV). The spectra were acquired in the constant-pass-energy mode using pass energies of 50 eV for survey spectra and 20 eV for high-resolution core-level spectra. The X-ray spot size was 650 μm, and the total experimental energy resolution was approximately 0.3 eV. Measurements were performed at room temperature under ultrahigh-vacuum conditions of approximately 1 × 10^−9^ mbar. An ion–electron charge compensation system was used to neutralize sample charging during spectral acquisition. The system was adjusted using polyethylene terephthalate (PET) reference samples according to the manufacturer’s recommendations. The binding-energy scale was calibrated using the Au 4f (84.0 eV), Ag 3d (368.2 eV), and Cu 2p (932.7 eV) reference peaks, with a calibration accuracy of 0.1 eV. Spectral processing and peak fitting were performed following the recommended practices described by Major et al. [31]. A Shirley background was subtracted from each high-resolution spectrum, and the spectral components were fitted using Gaussian functions. The same background-subtraction and peak-fitting procedure was applied to all spectra.

### 2.5. Adsorption Experiments

Stock solutions of Ni(II) and Co(II) with a concentration of 1000 mg/L, expressed as metal concentrations, were prepared by dissolving NiCl_2_·6H_2_O and CoCl_2_·6H_2_O, respectively, in distilled water. Working solutions with concentrations of 10–50 mg/L were prepared by diluting the stock solutions immediately before the experiments.

Adsorption experiments were conducted by adding 0.1 g of sorbent to 50 mL of a Ni(II) or Co(II) solution in a 250 mL flask. The suspensions were agitated on a PE-6410 orbital shaker (Ecroskhim LLC, St. Petersburg, Russia) at 150 rpm and room temperature. For equilibrium adsorption experiments, the initial metal-ion concentrations ranged from 10 to 50 mg/L, and the contact time was 24 h. Unless otherwise specified, the initial pH was not adjusted. The pH was measured before the addition of the sorbent and after completion of the experiment. Blank experiments without sorbent were performed under otherwise identical conditions. The effect of initial pH was examined for 10% CS/SiO_2_ and CS/SiO_2_–APTES at an initial metal-ion concentration of 10 mg/L and pH values of approximately 2.5 and 4.5, adjusted using dilute HCl. After adsorption, the supernatant was separated from the sorbent by centrifugation, and aliquots were collected for determination of the residual metal ion concentrations. For kinetic experiments, aliquots were collected at predetermined time intervals at an initial metal-ion concentration of 10 mg/L. After each sampling step, the remaining liquid phase and separated sorbent were returned to the flask. All adsorption experiments, including kinetic and isotherm experiments, were performed in triplicate (*n* = 3), and the results are presented as mean values ± standard deviation (SD).

The residual concentrations of Ni(II) and Co(II) were determined using an SF-2000 UV–Vis spectrophotometer (OKB Spektr, St. Petersburg, Russia). For analysis, the aliquots were diluted after the addition of the appropriate color-forming reagents to ensure that the metal concentrations were within the calibration range of the analytical method. Ni(II) was determined using dimethylglyoxime in the presence of sodium thiosulfate and potassium sodium tartrate at 460 nm [32], whereas Co(II) was determined using sodium diethyldithiocarbamate in an ammoniacal medium at 321 nm [33].

The removal efficiency (*R,* %) and equilibrium uptake (*q_e_*, mg/g) were calculated using the following equations [34]:

(1) $$R\;=\;\frac{C_{0}\;-\;C_{e}}{C_{0}}\;\times \;100\%$$

(2) $$q_{e}\;=\;\frac{C_{0}\;-\;C_{e}}{m}\;\times \;V$$

where *C*_0_ and *C_e_* (mg/L) are the initial and equilibrium metal-ion concentrations, respectively; *m* (g) is the weight of adsorbent used; *V* (L) is the volume of solution.

For kinetic experiments, the uptake at time *t* (*q_t_*, mg/g) was calculated from the metal-ion mass balance, accounting for the cumulative volume removed during sampling. The kinetic data were analyzed using the pseudo-first-order (PFO) and pseudo-second-order (PSO) models, expressed by Equations (3) and (4), respectively:

(3) $$q_{t}\;=\;q_{e}(1\;-\;e^{{-k}_{1}t})$$

(4) $$q_{t}\;=\;\frac{k_{2}q_{e}^{2}t}{1\;+\;k_{2}q_{e}t}$$

where *q_t_* and *q_e_* are the metal uptake values at time t and at equilibrium, respectively (mg/g); *k*_1_ is the PFO rate constant (min^−1^); *k*_2_ is the PSO rate constant (g·mg^−1^·min^−1^); and *t* is the contact time (min). The model parameters were determined by nonlinear fitting of the experimental data [35], and the goodness of fit was evaluated using the coefficient of determination (*R*^2^).

The equilibrium adsorption data were analyzed using the Langmuir and Freundlich models [36], expressed by Equations (5) and (6), respectively:

(5) $$q_{e}\;=\;\frac{q_{\max}K_{L}C_{e}}{1\;+\;K_{L}C_{e}}$$

(6) $$q_{e}\;=\;K_{F}C_{e}^{1/n}$$

where *q_e_* is the equilibrium metal uptake (mg/g), *C_e_* is the equilibrium metal-ion concentration (mg/L), *q_max_* is the model-derived maximum adsorption capacity parameter of the Langmuir equation (mg/g), *K_L_* is the Langmuir constant (L/mg), and *K_F_* and *n* are the Freundlich constants.

## 3. Results and Discussion

### 3.1. Characterization of the CS/SiO_2_ Composites

CS/SiO_2_ composites were prepared by precipitating chitosan from an acidic solution onto the silica surface through adjustment of the pH of a chitosan-silica suspension to 7.2–7.6. To prepare these mixed suspensions, calculated amounts of chitosan solution were added to a silica suspension to obtain final composites containing 5, 10, and 20 wt.% polymer. For convenience, these samples are hereafter denoted as 5% CS/SiO_2_, 10% CS/SiO_2_, and 20% CS/SiO_2_. Prior to alkalization, the upper part of the resulting suspensions appeared viscous and turbid. After adjusting the pH, a swollen curdy precipitate was formed, while the upper liquid phase became visually transparent. The amount of immobilized chitosan was determined from the difference between the polymer concentrations in the mother liquor before and after precipitation. The degree of chitosan immobilization and the calculated chitosan content in the composites are presented in Table 1.

The results demonstrated highly efficient deposition of chitosan onto the silica surface. For the 5% CS/SiO_2_ and 10% CS/SiO_2_ samples, the viscosity of the mother liquor was very close to that of pure water (0.97 ± 0.01 and 0.98 ± 0.005 mm^2^/s, respectively), indicating a very low residual chitosan content in the solution and an estimated immobilization degree close to 100%. For the 20% CS/SiO_2_ sample, the viscosity was also close to that of water but slightly higher (1.07 ± 0.01 mm^2^/s), corresponding to approximately 10 mg of residual chitosan in the solution and an estimated immobilization degree of approximately 96%. Consequently, the final chitosan contents in the composites were close to the target values and amounted to 5, 10, and 19 wt.% for the respective samples.

The presence and varying content of chitosan in the prepared CS/SiO_2_ composites were confirmed by thermogravimetric analysis (TGA) and Fourier Transform Infrared (FTIR) spectroscopy data.

**Figure 2:**
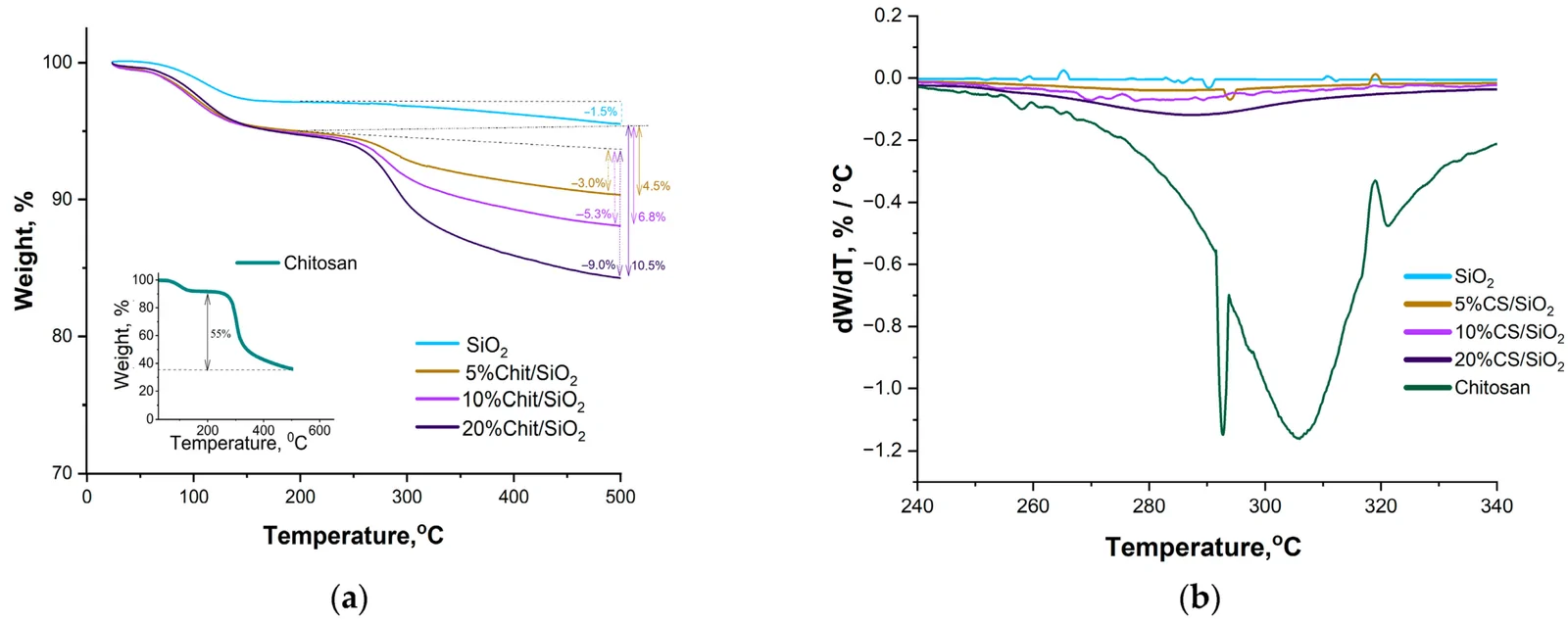
TGA **(a)** and DTG **(b)** curves of chitosan (inset), SiO_2_, and CS/SiO_2_ composites.

Figure 2 shows the TGA and DTG curves of SiO_2_, chitosan, and CS/SiO_2_ composites. All samples exhibit characteristic multistep thermal degradation behavior (Figure 2a). The thermal degradation of chitosan occurred in two stages. Below 200 °C, an initial weight loss of 8.3% was observed, corresponding to the removal of physically adsorbed water. The principal weight loss (55%) occurred in the temperature range of 250–500 °C and can be attributed to the pyrolytic degradation of chitosan with the release of volatile products (H_2_O, CO_2_, CO, NH_3_, CH_3_COOH, etc.) [37,38]. SiO_2_ also showed two stages of thermal degradation. The first stage, observed below 200 °C with a weight loss of 2.9%, is attributed to the removal of physically adsorbed water located on the surface and within the pore volume. A further mass loss of 1.5% in the range of 250–500 °C can be attributed to the release of bound water and partial thermal dehydration of surface silanol groups [39,40,41].

The CS/SiO_2_ composites exhibited a thermal behavior intermediate between those of pure chitosan and silica. The first weight-loss stage, observed below 200 °C, corresponded to the removal of physically adsorbed water. The mass loss in this region was similar for all composites (5.0–5.3%), being lower than that of pure chitosan (8.3%) but higher than that of SiO_2_ (2.9%). The second stage, occurring in the temperature range of 250–500 °C, can be attributed to the combined contribution of chitosan pyrolytic degradation and partial dehydration of the silica surface. The total mass losses in this region were 4.5%, 6.8%, and 10.5% for the 5% CS/SiO_2_, 10% CS/SiO_2_, and 20% CS/SiO_2_ composites, respectively. Taking into account that pure SiO_2_ exhibited a weight loss of 1.5% in the same temperature range the mass losses associated with chitosan decomposition were estimated to be approximately 3.0%, 5.3%, and 9.0%, respectively. Considering that pure chitosan loses 55% of its mass between 250 and 500 °C, the corresponding chitosan contents in the composites were calculated to be about 5, 9, and 16 wt.%, respectively. The DTG curves show that the second mass-loss stage occurs over a narrower temperature range in the composites than in pristine chitosan (Figure 2b). This difference indicates that immobilization on SiO_2_ affects the thermal decomposition behavior of chitosan. Nevertheless, the decomposition regions of both pristine and immobilized chitosan fall within the selected temperature range of 250–500 °C, supporting the use of this interval for estimating the chitosan-associated mass loss. Therefore, the chitosan contents estimated from TGA should be considered approximate.

The TGA-based chitosan contents were approximately 5, 9, and 16 wt.%, compared with 5, 10, and 19 wt.% estimated by the semiquantitative viscometric approach. The agreement was good at lower chitosan loadings, whereas a larger difference was observed for the 20% CS/SiO_2_ composite. Given the semiquantitative nature of both approaches, these values were used primarily to confirm the progressive increase in chitosan content across the composite series.

**Figure 3:**
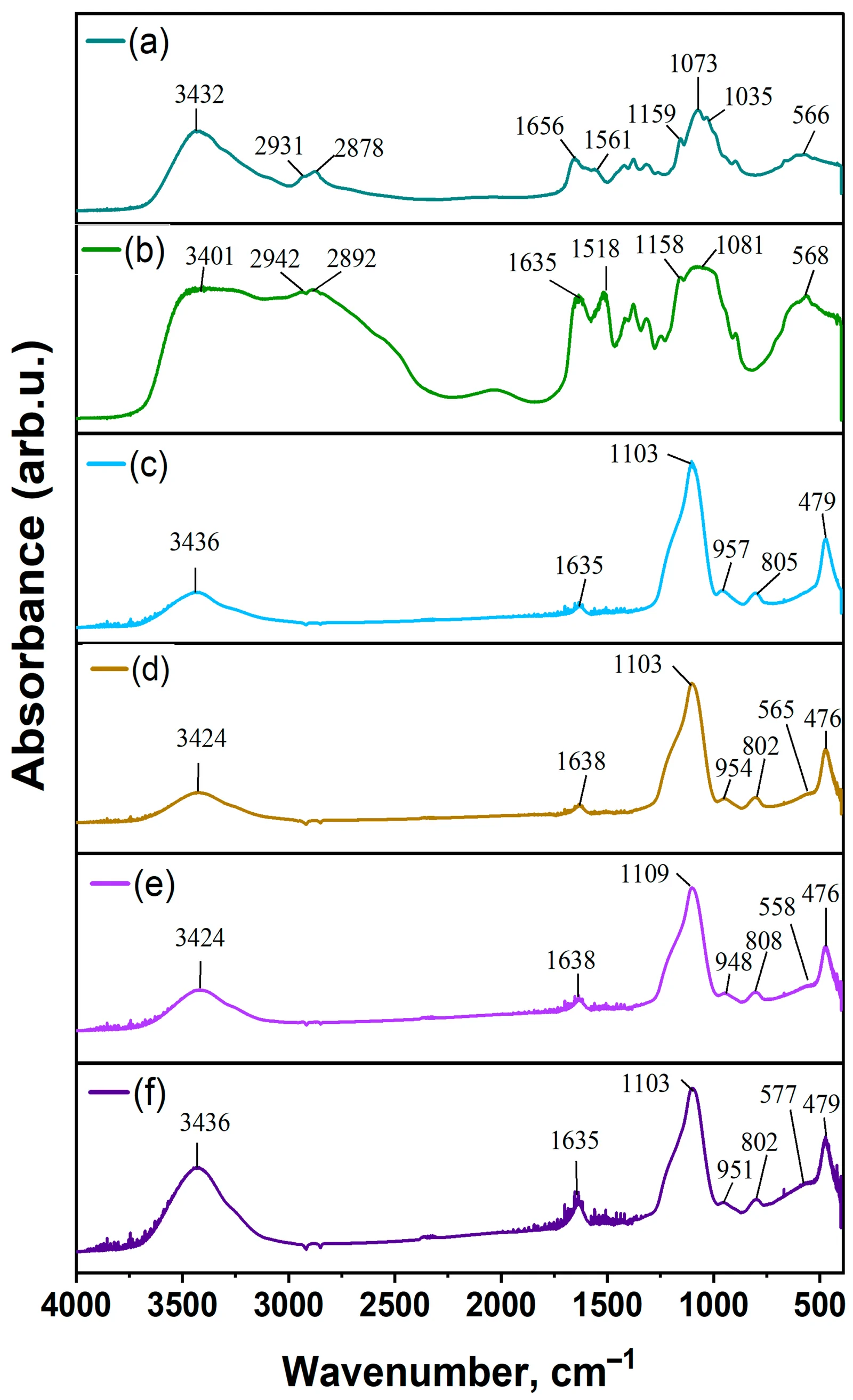
FTIR spectra of chitosan (**a**), chitosan hydrochloride (**b**), SiO_2_ (**c**), 5% CS/SiO_2_ (**d**), 10% CS/SiO_2_ (**e**) and 20% CS/SiO_2_ (**f**).

The FTIR spectroscopy results of chitosan, chitosan hydrochloride, and CS/SiO_2_ composites are presented in Figure 3. The FTIR spectrum of pristine chitosan exhibits a broad absorption band at 3432 cm^−1^ assigned to O–H and N–H stretching vibrations and bands at 2931 and 2878 cm^−1^ assigned to C–H stretching vibrations. The amide I and amide II bands are observed at 1656 and 1561 cm^−1^, respectively. The absorption bands in the 1000–1159 cm^−1^ region are attributed to C–O–C, C–OH, and C–C stretching vibrations of the saccharide ring structure. A band at 566 cm^−1^ is assigned to skeletal vibrations of the pyranose ring [42,43,44]. After dissolution in HCl, the characteristic absorption bands of chitosan shifted, particularly in the amide region. Two bands observed at 1635 and 1518 cm^−1^ are attributed to NH_3_^+^ groups, indicating protonation of the amino groups and the formation of chitosan hydrochloride [45].

The FTIR spectrum of pristine SiO_2_ exhibits a broad absorption band at 3436 cm^−1^, which is attributed to the stretching vibrations of surface hydroxyl groups and adsorbed water molecules. The band at 1635 cm^−1^ corresponds to the bending vibrations of silanol groups (Si–OH) and water molecules adsorbed on the silica surface. The characteristic absorption bands at 1103, 805, and 479 cm^−1^ are assigned to Si–O vibrations in the silica framework. In addition, the band at 957 cm^−1^ is attributed to the stretching vibrations of surface silanol (Si–OH) groups [46,47].

The FTIR spectra of the CS/SiO_2_ composites were generally similar to the spectrum of pristine SiO_2_, indicating that the silica framework remained unchanged after chitosan immobilization. Nevertheless, several spectral changes confirmed the presence of chitosan in the composites. In particular, the relative intensities of the broad band at 3424–3436 cm^−1^ and the band at 1635–1638 cm^−1^ increased with increasing chitosan content. Since the moisture contents of all composites were similar according to TGA data (5.0–5.3 wt.%), these changes can be attributed to the contribution of chitosan –OH and –NH functional groups. In addition, a weak band at 558–577 cm^−1^, assigned to skeletal vibrations of the pyranose ring of chitosan, appeared in the composite spectra and became more pronounced with increasing chitosan loading. Notably, the band at 1518 cm^−1^, characteristic of NH_3_^+^ groups in chitosan hydrochloride, was absent in the spectra of the CS/SiO_2_ composites, indicating deprotonation of the amino groups during composite preparation. A slight shift of the Si–OH band from 957 cm^−1^ in pristine SiO_2_ to 948–954 cm^−1^ in the composites was also observed, suggesting interactions between surface silanol groups and chitosan functional groups. The increased intensity of the band at 1635–1638 cm^−1^ may additionally reflect overlap with the amide I vibration of chitosan shifted as a result of interaction with the silica surface.

The effect of chitosan modification on morphological and textural properties of SiO_2_ was evaluated using nitrogen adsorption–desorption method and scanning electron microscopy (SEM).

**Figure 4:**
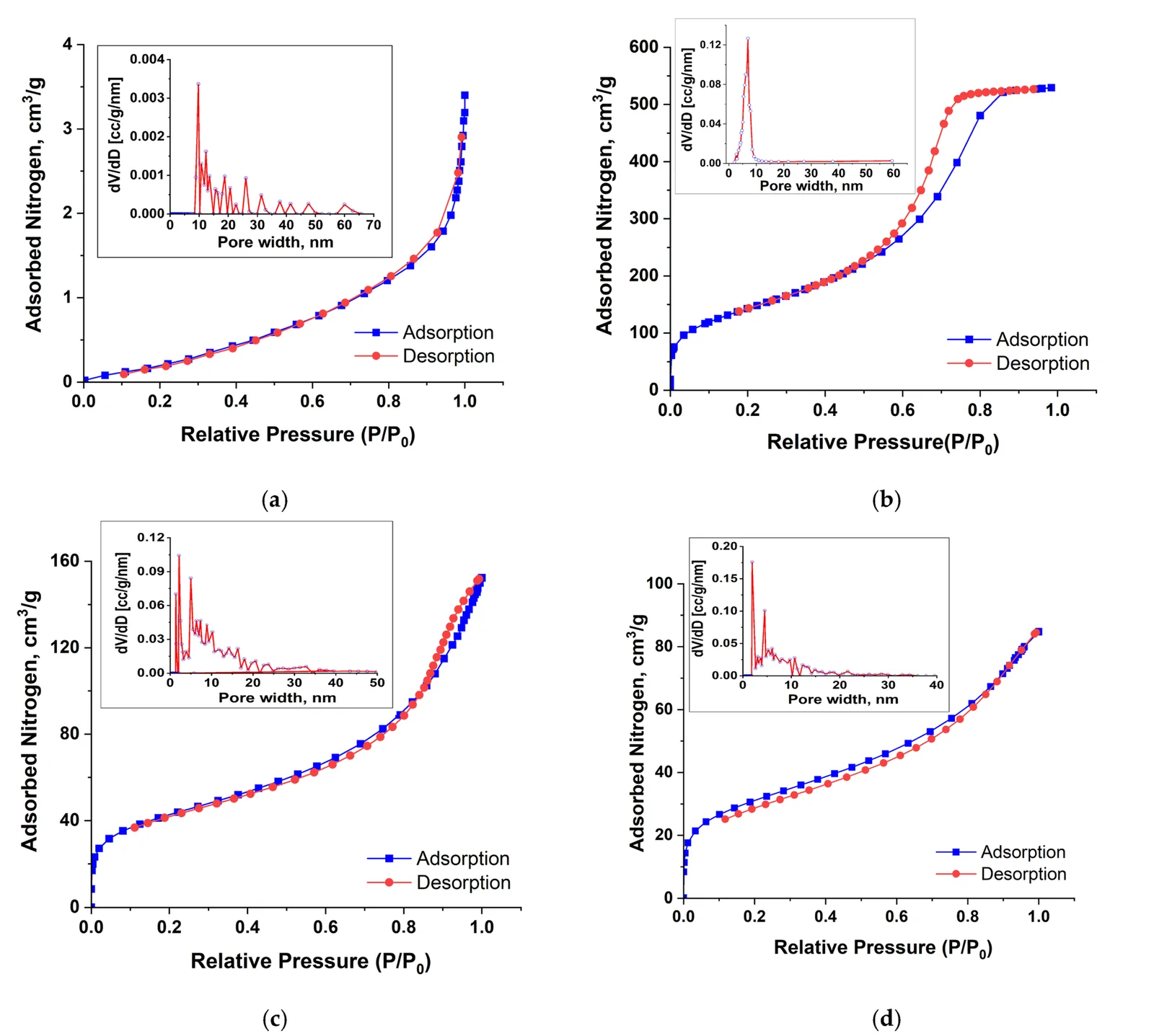

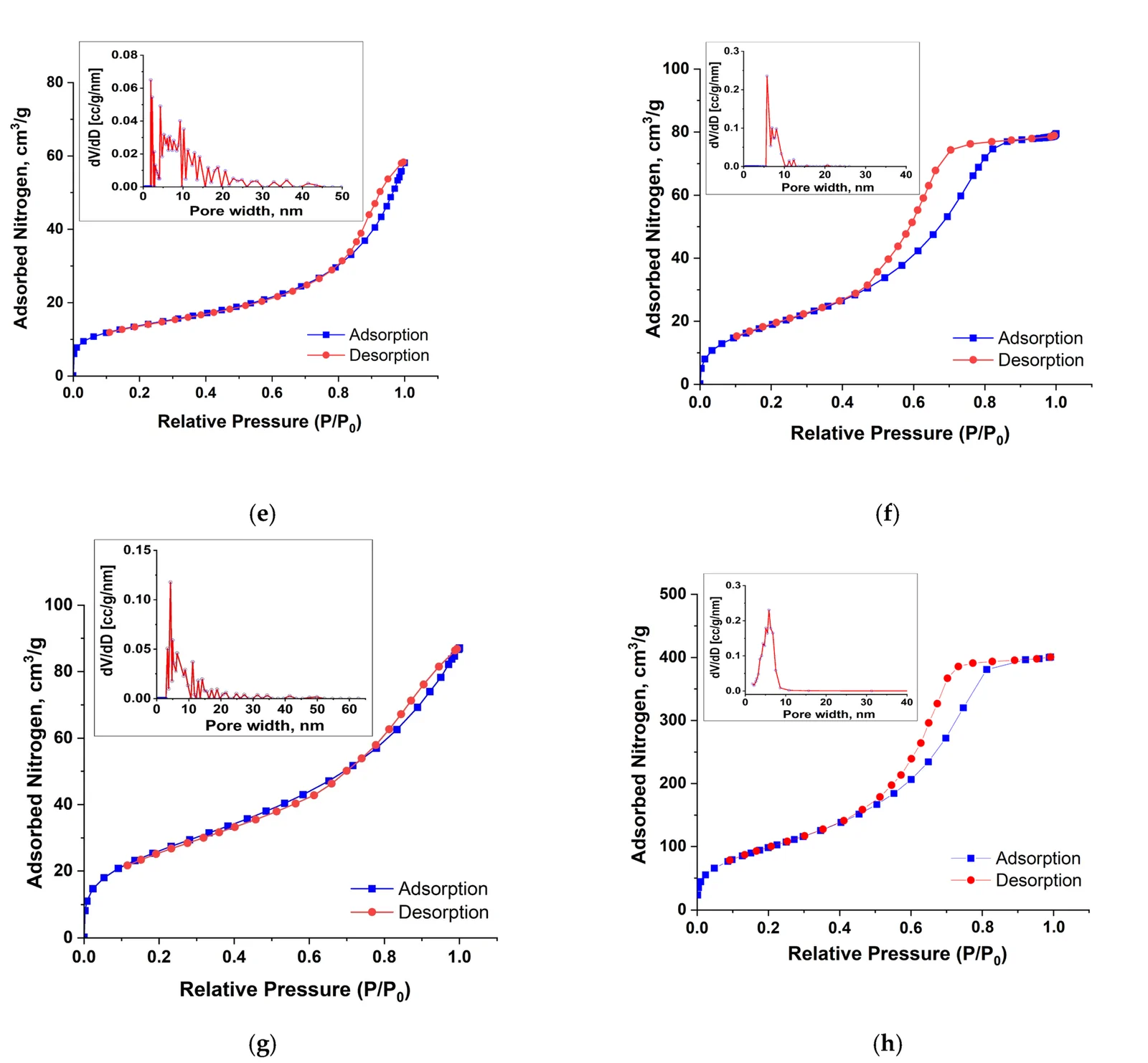
N_2_-adsorption−desorption isotherm plots of chitosan (**a**), SiO_2_ (**b**), 5% CS/SiO_2_ (**c**), 10% CS/SiO_2_ (**d**), 20% CS/SiO_2_ (**e**), SiO_2_–APTES (**f**), CS/SiO_2_–APTES (**g**), and SiO_2_–APTES-treated (**h**) composite. The insets show the corresponding pore-size distribution.

The N_2_ adsorption–desorption isotherm of chitosan (Figure 4a) belongs to type III of IUPAC classification, which is characteristic of non-porous or macroporous materials [48]. The low specific surface area (4.6 m^2^/g), together with the type III adsorption isotherm, indicates the absence of a well-developed micro- and mesoporous structure in chitosan. The isotherms for the SiO_2_ and CS/SiO_2_ composites were of type IV (Figure 4b–e), indicating that they are mesoporous materials [48].

The BJH pore size distribution curves revealed that pristine SiO_2_ possessed a relatively narrow pore size distribution, with a maximum centered at approximately 7 nm (Figure 4b, inset). In contrast, the CS/SiO_2_ composites (Figure 4c–e, insets) exhibited broader pore size distributions extending from 4 to 20 nm. These modifications of the pore architecture were accompanied by a gradual decrease in both the specific surface area and total pore volume with increasing chitosan content (Table 2). The observed decrease in the textural parameters can be attributed to the deposition of chitosan on the silica surface. This interpretation is supported by the slight shift of the Si–OH band observed in the FTIR spectra (Figure 3), indicating interactions between surface silanol groups and chitosan functional groups. As a result, partial coverage of the silica surface and blockage of smaller mesopores are likely to occur. Nevertheless, the type IV adsorption–desorption isotherms observed for all composites confirm that they retain a mesoporous structure after chitosan modification. The combination of preserved mesoporosity and the introduction of amino- and hydroxyl-containing functional groups may be beneficial for the adsorption of Ni(II) and Co(II) ions.

**Figure 5:**
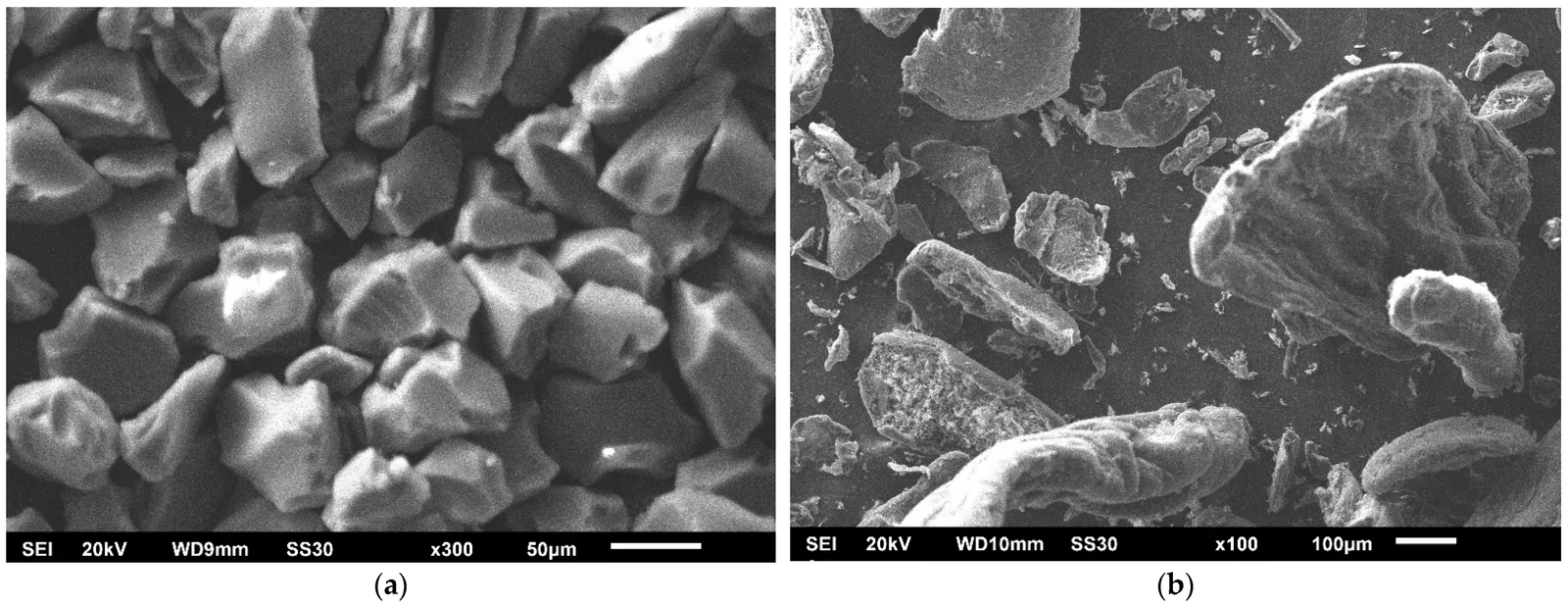

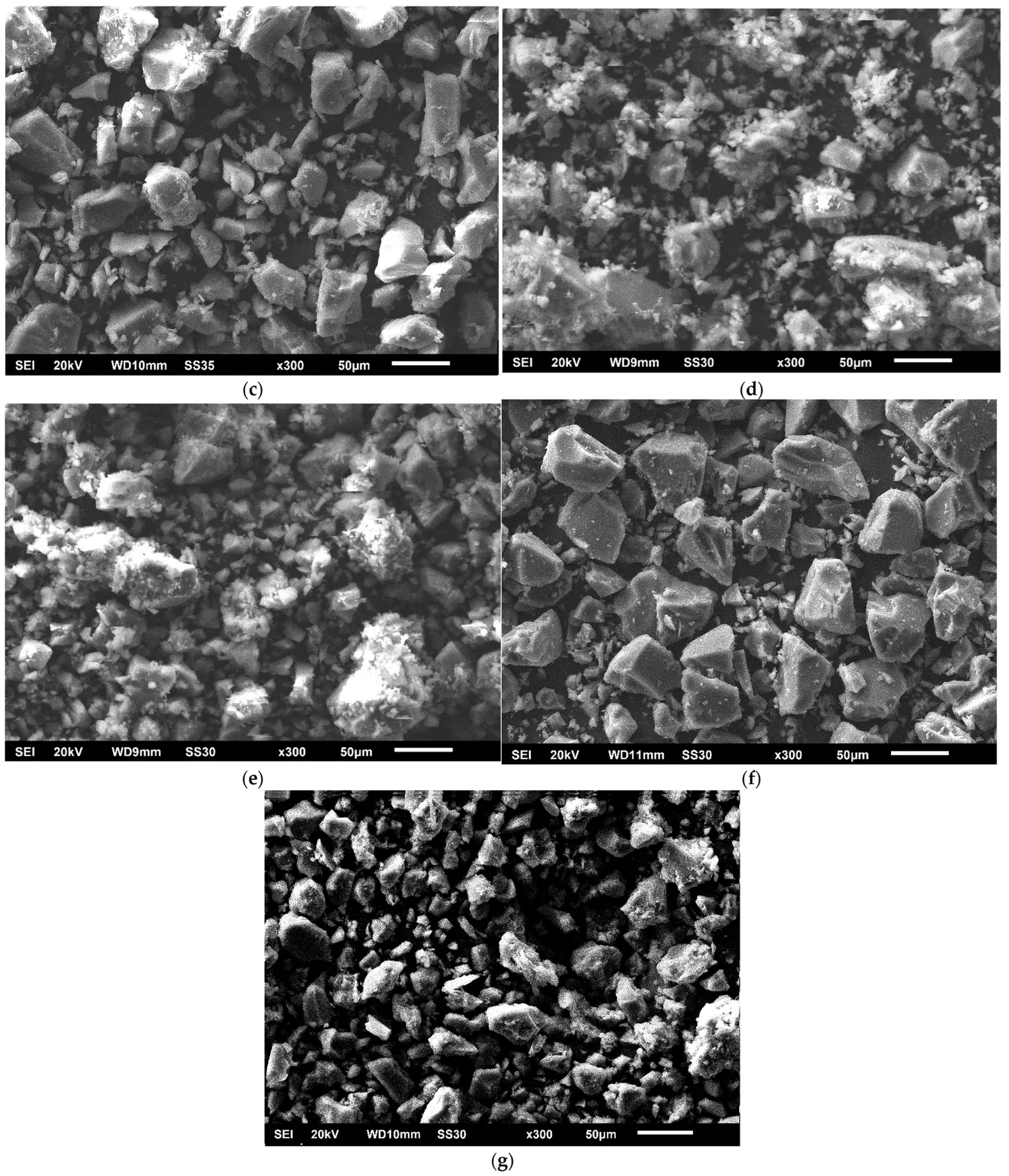
SEM microphotographs of SiO_2_ (**a**), chitosan (**b**), 5% CS/SiO_2_ (**c**), 10% CS/SiO_2_ (**d**), 20% CS/SiO_2_ (**e**), SiO_2_–APTES (**f**) and CS/SiO_2_–APTES (**g**).

SEM images revealed changes in the surface morphology of SiO_2_ after chitosan immobilization (Figure 5). The SEM image of pristine SiO_2_ (Figure 5a) reveals compact agglomerates of silica particles with sizes of approximately 50–100 μm. The particles exhibit relatively uniform morphology, irregular polyhedral shapes, and well-defined boundaries, with an apparently smooth and compact surface, which is typical for silica materials. SEM images of pristine chitosan (Figure 5b) reveal irregular flake-like agglomerates with relatively smooth faces. Although the morphology is heterogeneous, local folds, fibrous features, and minor surface irregularities characteristic of dried chitosan are observed. After immobilization of chitosan onto silica, the composites exhibited a morphology distinct from those of both pristine SiO_2_ and chitosan (Figure 5c–e). The large plate-like agglomerates characteristic of pristine chitosan were no longer observed. Instead, smaller irregular polymer domains were distributed over the silica surface, while regions retaining the smooth morphology of pristine SiO_2_ remained visible, indicating incomplete surface coverage. At the lowest chitosan loading of 5 wt.%, regions retaining the morphology of pristine SiO_2_ were particularly evident (Figure 5c). Increasing the chitosan loading to 10 wt.% resulted in more pronounced polymer-containing regions on the silica surface (Figure 5d). Only minor morphological differences were observed between the composites containing 10 and 20 wt.% chitosan (Figure 5e). Thus, within the investigated loading range, direct precipitation of chitosan onto SiO_2_ did not result in complete coverage of the silica surface, even at the highest polymer loading. These observations prompted us to investigate an alternative immobilization strategy involving preliminary functionalization of the silica surface with 3-aminopropyl triethoxysilane (APTES).

### 3.2. Characterization of the CS/SiO_2_–APTES Composite

To evaluate the effect of preliminary silica functionalization on chitosan immobilization, silica was first silanized in an ethanol medium using APTES. Chitosan was subsequently immobilized onto the modified silica (SiO_2_–APTES) using the same precipitation procedure as that employed for the preparation of the 10% CS/SiO_2_ composite. After neutralization, the mother liquor exhibited a viscosity comparable to that of water, indicating the absence of a noticeable amount of dissolved chitosan in the liquid phase. The residual chitosan concentration determined by viscometry corresponded to a chitosan content of approximately 10 wt.% in the obtained composite. The SiO_2_–APTES support and the resulting CS/SiO_2_–APTES composite were then characterized by FTIR spectroscopy, TGA, nitrogen adsorption–desorption, and SEM.

The FTIR spectra of the investigated samples are presented in Figure S1. The spectrum of SiO_2_–APTES (Figure S1a) exhibits a broad absorption band centered at approximately 3425 cm^−1^, attributed to O–H stretching vibrations of surface silanol groups and adsorbed water, with a possible contribution from N–H stretching vibrations of the grafted aminopropyl moieties. The bands at 1631 and 1501 cm^−1^ are associated with deformation vibrations of the APTES amino groups [49]. However, the band at approximately 1631 cm^−1^ may also contain a contribution from the bending vibrations of adsorbed water molecules. The presence of bands associated with the aminopropyl moieties confirms the functionalization of the SiO_2_ surface with APTES. Following chitosan immobilization, the overall appearance of the CS/SiO_2_–APTES spectrum (Figure S1b) changed only slightly. The most noticeable changes occurred in the 1400–1700 cm^−1^ region and involved changes in the relative intensities of the bands at approximately 1500 and 1640 cm^−1^. In the spectrum of CS/SiO_2_–APTES, the band at 1640 cm^−1^ became more pronounced relative to the band at 1507 cm^−1^. This may be related to the overlap of bands arising from N–H bending vibrations of the chitosan functional groups with those of the APTES aminopropyl moieties. However, the change in the relative intensities of these bands may be caused not only by the presence of chitosan but also by the partial removal of the grafted APTES layer during composite preparation. During chitosan deposition, the functionalized support was first exposed to an acidic chitosan solution and subsequently to a NaOH solution used to increase the pH, precipitate chitosan, and deprotonate its amino groups. Such acid-base treatment could result in the partial leaching of aminopropyl moieties from the support surface. To evaluate the effect of the acid-base treatment, an additional control sample, denoted SiO_2_–APTES-treated, was prepared. It was subjected to the same preparation conditions as CS/SiO_2_–APTES, but a hydrochloric acid solution of the same concentration was used instead of the chitosan solution, followed by neutralization with NaOH. The spectrum of the control sample (Figure S1c) showed a decrease in the relative intensity of the band at 1505 cm^−1^, which is consistent with the proposed partial removal of the aminopropyl moieties during the acid-base treatment. Simultaneously, the band at 1635 cm^−1^ became more pronounced, most likely because of an increase in the amount of adsorbed water. The spectra of SiO_2_–APTES-treated and CS/SiO_2_–APTES are generally very similar. The slight difference between the band positions observed at 1635 cm^−1^ for the control sample and at 1640 cm^−1^ for the chitosan-containing composite cannot be regarded as unambiguous evidence of the presence of chitosan because of the extensive overlap among the absorption bands of APTES, chitosan, and adsorbed water. Thus, the FTIR results do not allow the immobilization of chitosan on the functionalized surface to be reliably confirmed. Therefore, the compositions of the resulting samples were further investigated by thermogravimetric analysis.

The TGA curves of SiO_2_, SiO_2_–APTES, SiO_2_–APTES-treated, and CS/SiO_2_–APTES are presented in Figure S2. Pristine SiO_2_ was used as a reference to account for the weight loss associated with the inorganic support. Below 200 °C, all the investigated samples exhibited weight loss attributed primarily to the removal of physically adsorbed water. The most pronounced weight loss in this region was observed for SiO_2_–APTES-treated, which is consistent with the higher content of adsorbed water suggested by the FTIR results. In the temperature range of 200–350 °C, pristine SiO_2_ exhibited a weight loss of approximately 0.4 wt.%, increasing to approximately 2.9 wt.% at 900 °C relative to its weight at 200 °C. To account for this loss, a dashed baseline following the weight-loss profile of pristine SiO_2_ was drawn for each modified sample starting from its weight at 200 °C. The additional weight loss was determined as the difference between this baseline and the corresponding experimental curve. In the temperature range of 200–350 °C, the additional weight loss was approximately 1.6 wt.% for SiO_2_–APTES and 1.2 wt.% for SiO_2_–APTES-treated. A considerably higher value of approximately 5.4 wt.% was obtained for CS/SiO_2_–APTES, indicating an additional contribution from the thermal decomposition of immobilized chitosan. The chitosan content of CS/SiO_2_–APTES was estimated from the weight loss in the 200–350 °C range. After subtracting the contribution of the aminopropyl layer, estimated to be approximately 1.2–1.6 wt.%, the weight loss attributed primarily to chitosan decomposition was approximately 3.8–4.2 wt.%. Pristine chitosan loses approximately 44% of its weight within this temperature range. Accordingly, the chitosan content of CS/SiO_2_–APTES was estimated to be approximately 9–10 wt.%, in agreement with the viscometric result of approximately 10 wt.%. At 900 °C, the additional weight losses relative to the corresponding baselines were approximately 9.6 wt.% for SiO_2_–APTES, 5.2 wt.% for SiO_2_–APTES-treated, and 11.3 wt.% for CS/SiO_2_–APTES. The considerably lower weight loss of SiO_2_–APTES-treated compared with pristine SiO_2_–APTES indicates partial leaching of the aminopropyl moieties during exposure to HCl and subsequent neutralization with NaOH. The higher weight loss of CS/SiO_2_–APTES compared with the control sample results from the additional contribution of immobilized chitosan decomposition, together with the decomposition of the remaining aminopropyl layer [49]. Overall, the TGA results are consistent with the viscometric data and indicate a chitosan content of approximately 10 wt.% in CS/SiO_2_–APTES. The results also indicate partial leaching of the APTES layer during acid–base treatment.

Organic elemental analysis provided additional quantitative evidence for changes in the organic fraction during acid–base treatment (Table 3). The carbon content decreased from 10.5 wt.% in the original SiO_2_–APTES to 5.5 wt.% in SiO_2_–APTES-treated, while the hydrogen content decreased from 2.4 to 1.4 wt.%. This approximately twofold decrease is consistent with the reduction in the APTES-derived organic fraction observed by TGA. CS/SiO_2_–APTES contained 10.4 wt.% carbon and 1.8 wt.% hydrogen. Although its carbon content was close to that of the original SiO_2_–APTES, TGA confirmed the additional presence of approximately 10 wt.% immobilized chitosan in this material. Therefore, the similar overall organic content does not indicate complete retention of the APTES-derived phase and suggests that partial loss of APTES also occurs during chitosan immobilization under acidic conditions followed by neutralization.

The nitrogen adsorption–desorption isotherm of SiO_2_–APTES (Figure 4f) retained a shape similar to that of pristine SiO_2_ (Figure 4b). The corresponding pore-size distribution of SiO_2_–APTES (Figure 4f, inset) was slightly broader than that of SiO_2_ (Figure 4b, inset), although it remained considerably narrower than those of the CS/SiO_2_ composites (Figure 4c–e, insets). This suggests that APTES functionalization had a less pronounced effect on the pore-size distribution than chitosan modification. Despite these relatively minor changes in the pore-size distribution, APTES functionalization resulted in a substantial decrease in both the specific surface area and total pore volume (Table 2). Following chitosan immobilization, the adsorption–desorption isotherm and pore-size distribution of the 10 wt.% CS/SiO_2_–APTES (Figure 4g) composite became similar to those observed for the previously described CS/SiO_2_ composites (Figure 4c–e), which is consistent with the presence of the polymer in the modified material. Notably, the BET surface area of CS/SiO_2_–APTES (265.9 m^2^/g) was higher than that of the original SiO_2_–APTES (219.9 m^2^/g), despite the additional incorporation of chitosan. This increase is consistent with partial loss of the APTES-derived organic layer during chitosan immobilization under acidic conditions followed by neutralization, as also suggested by TGA and elemental analysis.

The SiO_2_–APTES-treated control further supports this interpretation. After acid–base treatment, the BET surface area and total pore volume increased from 219.9 to 370.8 m^2^/g and from 0.373 to 0.619 cm^3^/g, respectively, while remaining below those of pristine SiO_2_. At the same time, the adsorption–desorption isotherm and pore-size distribution became closer to those of pristine SiO_2_, indicating partial recovery of the original textural characteristics of the silica support (Figure 4h). These changes are consistent with the partial removal of the APTES-derived organic layer during treatment.

SEM observations provide further evidence that preliminary APTES functionalization affects the morphology of the resulting chitosan-containing composite (Figure 5). The SiO_2_–APTES sample (Figure 5f) retained a particle morphology generally similar to that of pristine SiO_2_ (Figure 5a). Clear morphological changes were observed after chitosan immobilization (Figure 5g). In the directly prepared 10 wt.% CS/SiO_2_ composite (Figure 5d), distinct silica particles coexisted with more dispersed, irregular regions of different morphology, which can be attributed to the polymer phase. In contrast, such distinct polymer-like domains were largely absent in 10 wt.% CS/SiO_2_–APTES. Instead, the composite exhibited a more uniform morphology, while the surface appearance of the particles differed from that of SiO_2_–APTES. Together with the TGA evidence for the presence of approximately 10 wt.% immobilized chitosan, these observations suggest that the immobilization strategy influences the morphology and organization of the polymer-containing phase. The more uniform surface appearance of CS/SiO_2_–APTES is consistent with a potentially more homogeneous distribution of chitosan, although its spatial distribution cannot be directly established from SEM observations alone.

### 3.3. Adsorption Properties Toward Ni(II) and Co(II)

The adsorption properties of the prepared materials toward Ni(II) and Co(II) were evaluated to examine how chitosan loading and the immobilization strategy affect metal-ion uptake. The adsorption behavior was first compared at an initial metal ion concentration of 10 mg/L, followed by kinetic studies at the same concentration and concentration-dependent uptake experiments over the range of 10–50 mg/L. The effect of chitosan loading was first examined for the directly prepared CS/SiO_2_ composites, followed by evaluation of the influence of preliminary APTES functionalization.

The initial pH of the metal-containing solutions was not adjusted and generally ranged from 6.3 to 6.8. Control experiments without adsorbent were performed under the same conditions. After 24 h, a slight pH drift was observed in the blank solutions (6.6–7.0), while the measured Ni(II) and Co(II) concentrations remained close to their initial values.

#### 3.3.1. Effect of Chitosan Loading

**Figure 6:**
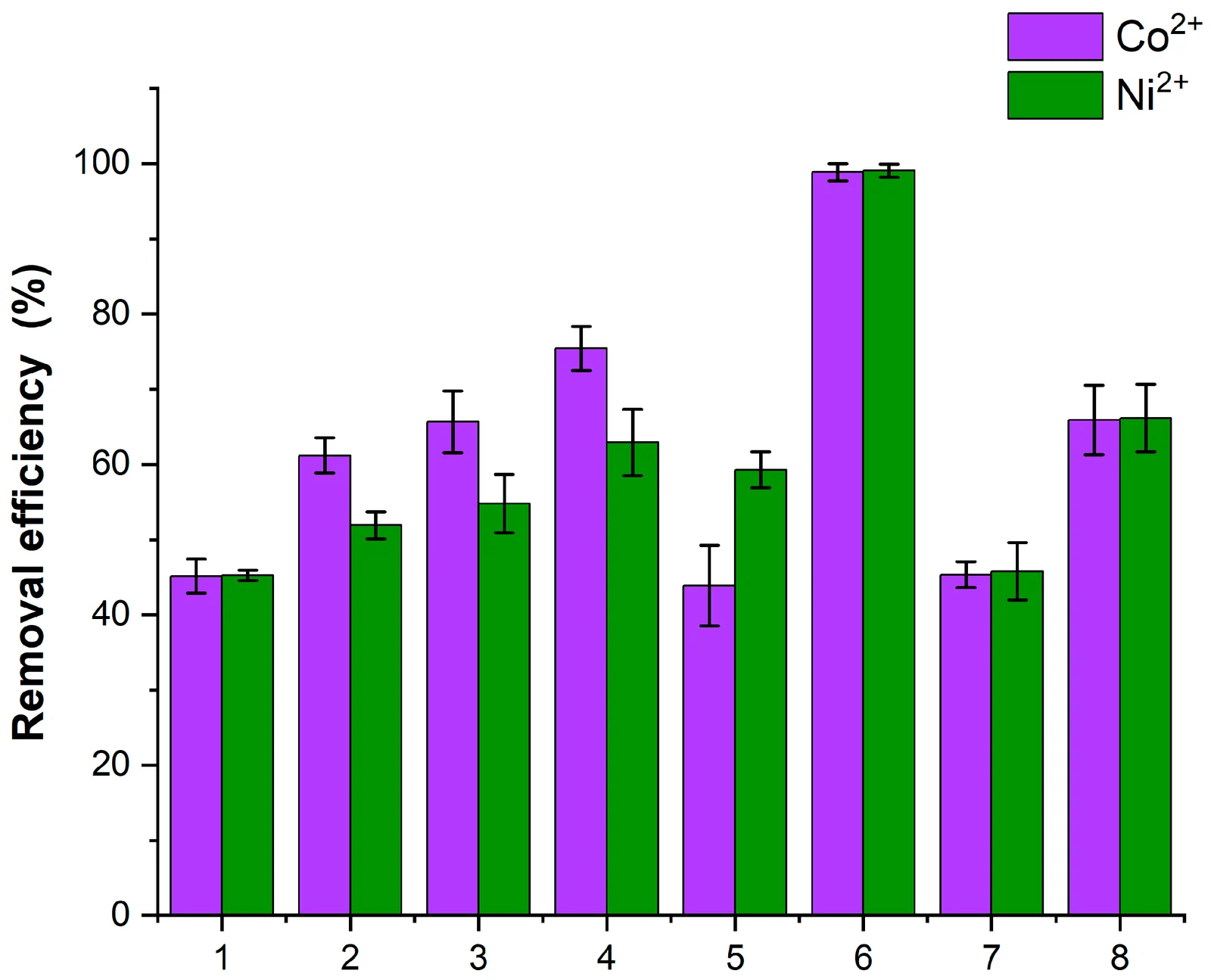
Removal efficiency of Ni(II) and Co(II) ions by different sorbents: SiO_2_ (1); 5% CS/SiO_2_ (2); 10% CS/SiO_2_ (3); 20% CS/SiO_2_ (4); chitosan (5); SiO_2_–APTES (6); SiO_2_–APTES–treated (7); and CS/SiO_2_–APTES (8).

The effect of chitosan loading on the removal of Ni(II) and Co(II) was evaluated at an initial metal ion concentration of 10 mg/L by comparing the removal efficiencies of the prepared materials (Figure 6).

Chitosan modification improved the adsorption performance of SiO_2_ toward both metal ions, with a noticeable effect already observed at a relatively low polymer loading of 5 wt.%. For Co(II), the removal efficiency increased from 45% for pristine SiO_2_ to 61% for the 5 wt.% CS/SiO_2_ composite and further to 75% for the 20 wt.% composite. For Ni(II), the improvement was less pronounced, with the removal efficiency increasing from 45% for pristine SiO_2_ to 52% and 63% for the 5 and 20 wt.% CS/SiO_2_ composites, respectively.

Notably, the improvement in adsorption upon chitosan modification occurred despite the decrease in the BET surface area and total pore volume of the composites (Table 2). This suggests a positive contribution of the introduced chitosan phase to metal-ion removal, consistent with the well-established ability of chitosan amino groups to bind metal cations [50].

SEM observations provide complementary information on the organization of the chitosan phase. As described above, immobilization on SiO_2_ resulted in a considerably more dispersed polymer phase compared with pristine chitosan, while increasing the polymer loading did not lead to complete coverage of the silica surface (Figure 5).

Chitosan is known to form an extensive hydrogen-bonding network involving hydroxyl and amino groups, which promotes interactions between polymer chains [51,52]. The FTIR results (Figure 3) additionally indicate interactions between chitosan functional groups and surface silanol groups in the composites. These interactions may contribute to the more dispersed state of chitosan observed after its immobilization on SiO_2_. The difference in the degree of chitosan dispersion may therefore affect the accessibility of its amino groups for metal-ion binding.

To obtain a quantitative comparative estimate of the effective utilization of chitosan amino groups, metal uptake was normalized to the estimated amount of chitosan-derived nitrogen. The contribution of the silica support was taken into account because pristine SiO_2_ itself showed appreciable removal of Ni(II) and Co(II) under the investigated conditions (calculation details are provided in Section S1 and Table S1 of the Supplementary Materials). The calculated N-normalized uptake was consistently higher for all three supported chitosan composites than for pristine chitosan. This trend is consistent with the SEM observations showing a more dispersed polymer-containing phase after immobilization on SiO_2_, suggesting that polymer dispersion may contribute to more effective utilization of nitrogen-containing functional groups. However, the calculated values are approximate and should not be regarded as direct measurements of amino-group accessibility.

Overall, the results indicate that increasing chitosan loading does not lead to a proportional improvement in Ni(II) and Co(II) uptake. At the same time, the observed morphological differences and the higher calculated N-normalized uptake of the supported composites suggest that the organization of the polymer-containing phase may influence metal uptake in addition to the overall chitosan content. Therefore, an alternative immobilization strategy involving preliminary functionalization of SiO_2_ with APTES was further examined.

#### 3.3.2. Effect of the Immobilization Strategy

The effect of the immobilization strategy was further evaluated at the same initial metal ion concentration of 10 mg/L. Since the CS/SiO_2_–APTES composite contained approximately 10 wt.% chitosan, the directly prepared 10 wt.% CS/SiO_2_ composite was used as a reference, allowing the two immobilization approaches to be compared at a similar polymer loading.

Preliminary APTES functionalization had a pronounced effect on the adsorption properties of the silica support. SiO_2_–APTES achieved nearly complete removal of both Ni(II) and Co(II), substantially exceeding the removal efficiencies observed for pristine SiO_2_. In contrast, the removal efficiencies of the SiO_2_–APTES-treated control decreased markedly to approximately 46% for Ni(II) and 45% for Co(II). This decrease was accompanied by an approximately twofold reduction in the APTES-derived organic fraction, as indicated by elemental analysis (C: 10.5 to 5.5 wt.%) and TGA (9.6 to 5.2 wt.%, Figure S2). At the same time, the BET surface area partially recovered from 219.9 to 370.8 m^2^/g, compared with 517.9 m^2^/g for pristine SiO_2_ (Table 2).

Chitosan immobilization on SiO_2_–APTES led to an increase in the organic content of the resulting material. According to TGA and elemental analysis, the organic content of CS/SiO_2_–APTES was approximately twice that of the SiO_2_–APTES-treated control and comparable to that of the original SiO_2_–APTES. The removal efficiencies of CS/SiO_2_–APTES reached approximately 66% for Ni(II) and 65% for Co(II), exceeding the corresponding values obtained for the treated control. However, CS/SiO_2_–APTES remained substantially less effective than the original SiO_2_–APTES, despite their similar overall organic contents. Notably, the final solution pH after metal adsorption was also higher for the original SiO_2_–APTES (8.0–8.6) than for SiO_2_–APTES-treated (7.8–8.4) and CS/SiO_2_–APTES (7.6–8.2). At these elevated pH values, possible contributions from metal-ion hydrolysis cannot be excluded. These observations suggest that metal-ion removal is influenced not only by the total organic content but also by the nature and state of the corresponding surface functional groups.

The two chitosan-containing composites differed in their textural and morphological characteristics despite their similar chitosan contents. CS/SiO_2_–APTES exhibited a lower BET surface area than directly prepared 10% CS/SiO_2_ (Table 2). SEM observations also revealed differences in the morphology and apparent distribution of the polymer-containing phase. In directly prepared 10% CS/SiO_2_, discrete irregular polymer domains were visible on the silica particles, whereas such distinct domains were not observed for CS/SiO_2_–APTES (Figure 5). Despite these structural differences, both composites showed relatively similar removal efficiencies for Ni(II) and Co(II), with somewhat higher Ni(II) removal observed for CS/SiO_2_–APTES.

**Figure 7:**
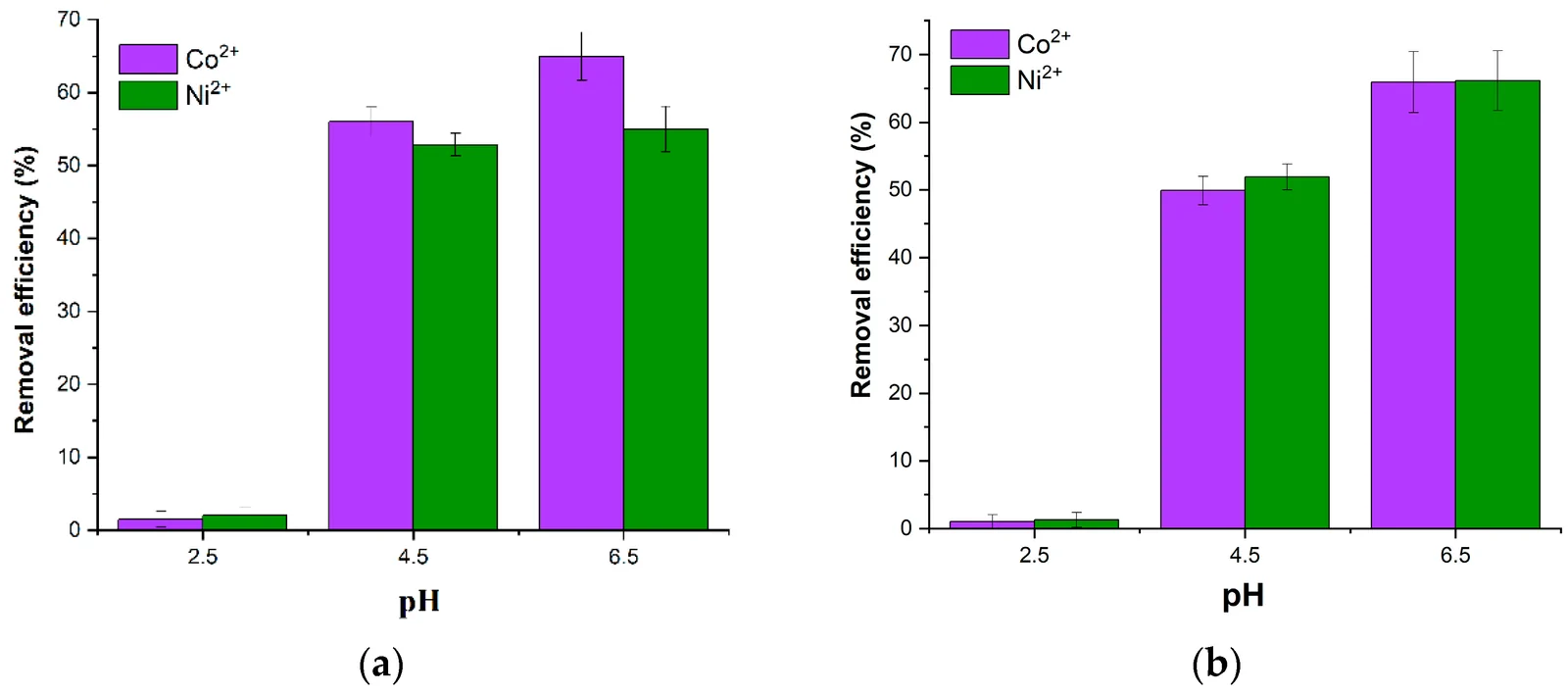
Effect of initial solution pH on Ni(II) and Co(II) removal by 10% CS/SiO_2_ (**a**) and CS/SiO_2_–APTES (**b**) composites.

To further compare the adsorption behavior of the two chitosan-containing composites prepared using different immobilization strategies, the effect of initial solution pH on Ni(II) and Co(II) removal was investigated (Figure 7).

At an initial pH of approximately 2.5, both composites exhibited negligible metal removal, while the final solution pH remained close to its initial value (2.7–2.8). At an initial pH of approximately 4.5, the removal efficiencies were only moderately lower than those obtained without pH adjustment. This relatively limited decrease may be associated with the substantial increase in solution pH during contact with the sorbents, reaching 7.6–7.8, which is comparable to the final pH values observed without initial pH adjustment (7.7–8.2). Notably, under the conditions without initial pH adjustment, the final pH values of the blank solutions (6.6–7.0) and those containing pristine SiO_2_ were relatively close (6.8–7.2), whereas the chitosan-containing composites exhibited higher values. These observations are consistent with differences in the acid–base behavior of the materials following surface modification, although they do not directly establish the surface charge or protonation state of individual functional groups.

Preliminary regeneration experiments were also performed using 0.1 M HCl for metal desorption. However, acid treatment caused swelling of the chitosan-containing composites, making quantitative recovery of the solid sorbents by filtration or centrifugation difficult. Consequently, further adsorption–desorption cycles could not be reliably performed. These observations indicate limitations of the investigated composites under acidic regeneration conditions.

#### 3.3.3. Adsorption Isotherms and Kinetics

**Figure 8:**
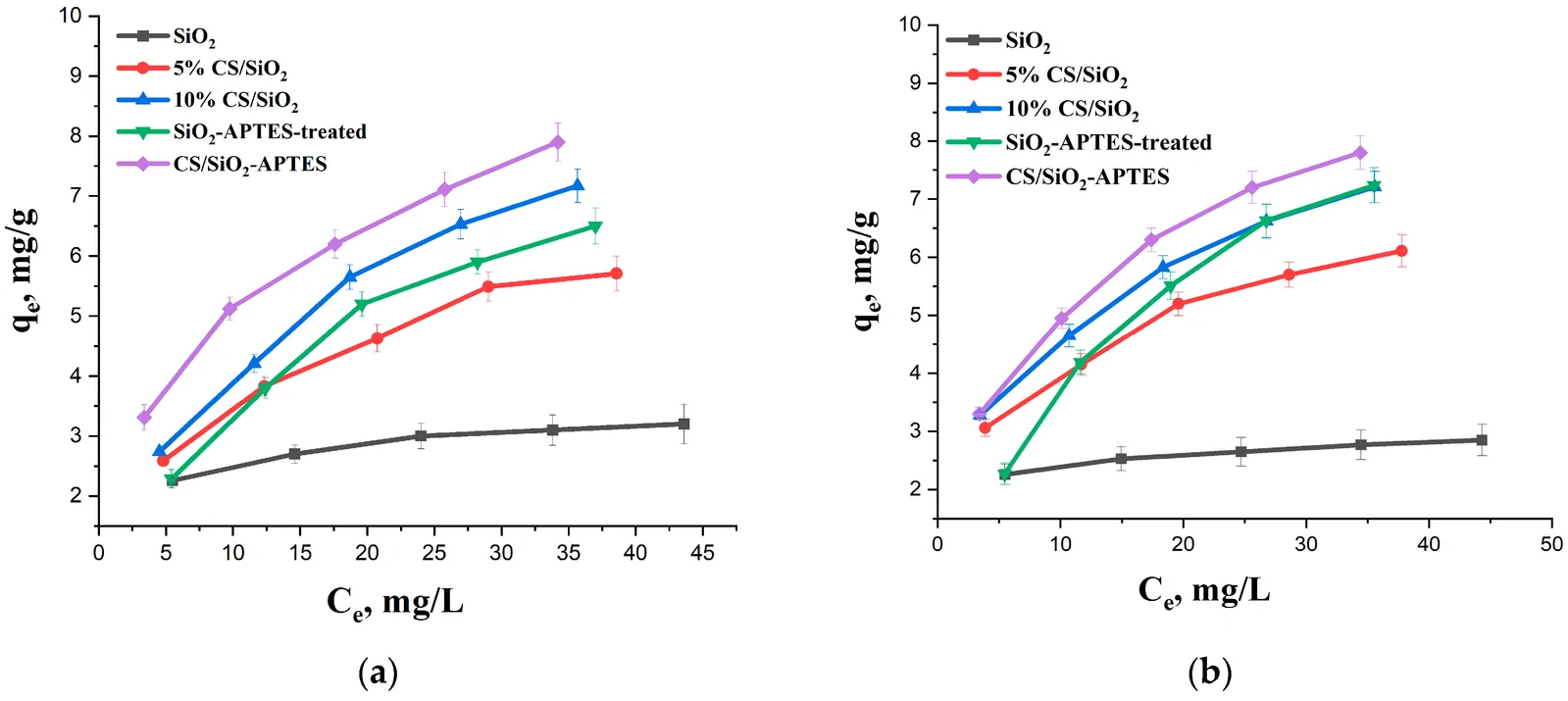
Adsorption isotherms of Ni(II) (**a**) and Co(II) (**b**) on the selected sorbents.

To further evaluate the effects of chitosan loading and immobilization strategy on adsorption behavior over a wider concentration range, five representative materials (SiO_2_, 5% CS/SiO_2_, 10% CS/SiO_2_, SiO_2_–APTES-treated, and CS/SiO_2_–APTES) were selected for further investigation. The corresponding Ni(II) and Co(II) adsorption isotherms are presented in Figure 8.

For both metal ions, the equilibrium uptake increased with increasing equilibrium concentration, although the magnitude of this increase varied among the investigated materials. Pristine SiO_2_ exhibited a relatively small increase in metal uptake, whereas the chitosan-containing composites and SiO_2_–APTES-treated showed a more pronounced increase. This behavior is consistent with changes in the adsorption properties of the silica surface following modification. Notably, these modified materials also exhibited higher final solution pH values (generally around 7.7–8.4) than pristine SiO_2_ (6.8–7.2), whose final pH remained relatively close to that of the blank solutions (6.6–7.0). These observations suggest that differences in concentration-dependent metal uptake are accompanied by changes in the final solution pH following surface modification. Therefore, the calculated equilibrium uptake values (q_e_) may include contributions from adsorption as well as pH-dependent hydrolysis and possible surface precipitation. The relative contributions of these processes cannot be distinguished from the present experimental data. Comparison of the three chitosan-containing composites revealed some differences in Ni(II) and Co(II) uptake. However, their equilibrium uptake values remained within a relatively similar range throughout the investigated concentrations. Despite the differences in composition and the morphological variations observed by SEM, the composites exhibited no substantial differences in metal uptake under the investigated conditions.

The experimental adsorption data were analyzed using the linearized Langmuir and Freundlich isotherm models (Table S2). Both models generally exhibited high coefficients of determination in their respective linearized forms. For the three chitosan-containing composites, slightly higher values were obtained using the Freundlich model, which may be consistent with the presence of adsorption sites with different chemical environments. In the directly prepared CS/SiO_2_ composites, SEM observations revealed polymer-containing domains alongside regions retaining the morphology of pristine silica. In contrast, CS/SiO_2_–APTES exhibited a more homogeneous surface morphology, although chemically different surface functional groups may still be present. However, the isotherm modeling results alone do not establish the nature of metal-ion binding or a specific adsorption mechanism.

**Figure 9:**
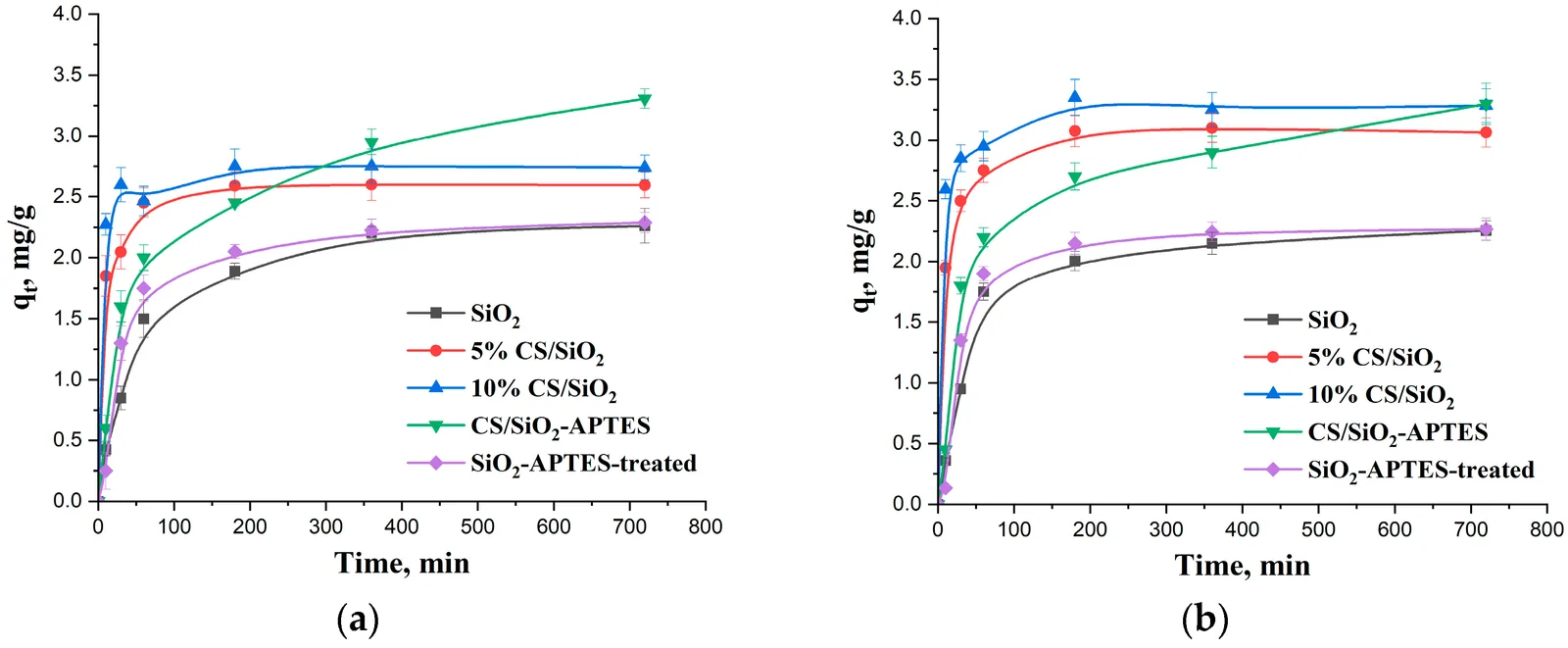
Adsorption kinetics of Ni(II) (**a**) and Co(II) (**b**) on the selected sorbents.

The adsorption kinetics of Ni(II) and Co(II) were investigated for the five selected materials at an initial metal-ion concentration of 10 mg/L (Figure 9). The directly prepared CS/SiO_2_ composites exhibited relatively rapid initial metal uptake, approaching stable values within the first hour. In contrast, CS/SiO_2_–APTES showed a more gradual increase in metal uptake. The observed differences in adsorption kinetics appear to be consistent with variations in composite morphology associated with the immobilization strategy, despite the relatively similar equilibrium metal uptake.

The experimental kinetic data were analyzed using pseudo-first-order (PFO) and pseudo-second-order (PSO) models (Table S3). For all three chitosan-containing composites, the PSO model provided a better description of both Ni(II) and Co(II) uptake, as indicated by higher coefficients of determination (R^2^). The model-calculated equilibrium uptake values were also in good agreement with the experimental values measured after 24 h. Nevertheless, agreement with the PSO model alone does not establish a chemisorption-controlled mechanism or identify the rate-limiting step of adsorption. Therefore, to further investigate possible interactions of Ni(II) and Co(II) with surface functional groups, the metal-loaded composites were characterized by FTIR spectroscopy and XPS.

### 3.4. Characterization of Ni(II)- and Co(II)-Loaded Composites

FTIR spectra of Ni(II)- and Co(II)-loaded CS/SiO_2_, CS/SiO_2_–APTES, and SiO_2_–APTES composites were compared with those of the corresponding initial materials (Figure S3a–c). For CS/SiO_2_, only minor changes were observed in the broad O–H/N–H stretching region (~3400 cm^−1^) and around 1635 cm^−1^ after metal adsorption. The band at 577 cm^−1^, previously associated with vibrations of the chitosan pyranose ring, shifted slightly to 574 and 568 cm^−1^ after Co(II) and Ni(II) adsorption, respectively. More pronounced changes were observed for the band at 951 cm^−1^, assigned to silanol-related vibrations, which shifted to 958 and 970 cm^−1^ after Co(II) and Ni(II) adsorption, respectively. This observation is consistent with the SEM results showing partially exposed silica regions in the directly prepared composite, which may remain accessible for interactions with metal ions.

In contrast, CS/SiO_2_–APTES exhibited only minor spectral changes after Ni(II) and Co(II) adsorption. The bands associated with the chitosan-containing phase showed no pronounced shifts, while the silanol-related band remained essentially unchanged at 951–952 cm^−1^. For SiO_2_–APTES, the corresponding band showed only minor shifts from 953 cm^−1^ to 951 and 949 cm^−1^ after Co(II) and Ni(II) adsorption, respectively. Thus, both APTES-modified materials exhibited negligible changes in the silanol-related region, in contrast to the more pronounced shifts observed for directly prepared CS/SiO_2_. This distinction is consistent with the differences in surface morphology observed by SEM, although a direct relationship between morphology and the spectral changes cannot be established. In the region associated with nitrogen-containing functionalities, a different trend was observed. For SiO_2_–APTES, the band at 1501 cm^−1^ shifted to 1508 and 1507 cm^−1^ after Co(II) and Ni(II) adsorption, respectively. In contrast, the corresponding weak spectral feature in CS/SiO_2_–APTES showed no clearly distinguishable changes. This difference may reflect changes in the local environment of APTES-derived functional groups after chitosan immobilization, although the weak and overlapping bands limit further interpretation.

Overall, the FTIR results reveal differences in the spectral response of the materials following metal adsorption, which are consistent with variations in their surface composition and morphology. However, the relatively minor changes in chitosan-associated bands do not exclude the involvement of chitosan functional groups in metal binding, particularly given the extensive overlap of characteristic vibrational bands.

To further investigate possible interactions between metal ions and nitrogen-containing surface groups, X-ray photoelectron spectroscopy (XPS) was used. The composites were analyzed before and after metal adsorption, focusing on the Co 2p, Ni 2p, and N 1s core-level spectra. The corresponding XPS spectra are presented in the Supplementary Information (Figures S4–S6), while the results of their analysis are summarized in Table 4 and Table 5.

In the spectra of the samples loaded with Co (II) ions, two peaks located at ~781.0–782.3 and ~796.2–797.9 eV can be attributed to the Co^2+^ 2*p*3/2 and Co^2+^ 2*p*1/2 components, respectively (Table 4). The presence of intense shake-up satellites located approximately 5–6 eV above the main Co 2*p*3/2 and Co 2*p*1/2 peaks is a characteristic feature of paramagnetic Co(II) compounds and further supports the presence of Co^2+^ on the surface of sorbents [53]. For the SiO_2_–Co sample, the Co 2*p*3/2 and Co 2*p*1/2 peaks were observed at 782.3 and 797.9 eV, respectively. These values are in good agreement with the literature data reported for Co(II) supported on SiO_2_ [53]. In the case of chitosan–Co, the Co 2*p*3/2 (781.0 eV) and Co 2*p*1/2 (796.2 eV) peaks were located at lower binding energies than those of SiO_2_–Co. A similar shift toward lower binding energies was previously observed for a Co(II)–chitosan film and was associated with an increase in the electron density around Co(II) resulting from electron donation from the O and N atoms of chitosan [54]. For the composite sorbents, the Co 2*p*3/2 and Co 2*p*1/2 peaks were also observed at lower binding energies than those for SiO_2_–Co, although the differences were less pronounced than for chitosan–Co. Similar trends in the binding energies were observed for the Ni-containing systems. In the spectra of the samples loaded with Ni ions, two peaks located at ~855.1–856.2 and ~872.7–874.3 eV, corresponding to the Ni 2*p*3/2 and Ni 2*p*1/2 components, respectively, were observed together with characteristic shake-up satellites (Table 4). The Ni 2*p*3/2 (856.2 eV) and Ni 2*p*1/2 (874.3 eV) peak positions observed for the SiO_2_–Ni sample were close to binding energies of 856.6 and 874.1 eV reported for Ni(II) species supported on SiO_2_ [55]. For chitosan–Ni, the Ni 2*p*3/2 (855.1 eV) and Ni 2*p*1/2 (872.7 eV) peaks were located at the lowest binding energies among the investigated Ni-containing samples. Similar to the Co-containing system, the Ni 2*p*3/2 and Ni 2*p*1/2 peaks of the CS/SiO_2_–Ni, SiO_2_–APTES–Ni, and CS/SiO_2_–APTES–Ni samples were observed at intermediate binding energies between those of SiO_2_–Ni and chitosan–Ni. Thus, the lower binding energies observed for Co and Ni in the composite sorbents compared with SiO_2_ may be associated with the involvement of amino groups of the organic components in metal-ion binding.

To further evaluate the possible involvement of nitrogen-containing functional groups in Co(II) and Ni(II) binding, the N 1s spectra of the composite sorbents before and after metal adsorption were compared (Figure S6, Table 5). The N 1s spectra exhibit an asymmetric shape, indicating the presence of nitrogen species in different chemical environments (Figure S6). Deconvolution of the N 1s spectra of the sorbents before metal adsorption revealed a main component at ~399–400 eV and a higher-binding-energy shoulder at ~401–402 eV. The main component corresponds to free amino groups (–NH_2_), while the higher-binding-energy component can be attributed to amide nitrogen and/or protonated amino groups (–NH_3_^+^) [56,57]. After Co(II) and Ni(II) adsorption, the main N 1s component shifted toward higher binding energies in all three composite systems (Table 5). The shifts were most pronounced for CS/SiO_2_, whereas smaller changes were observed for SiO_2_–APTES and CS/SiO_2_–APTES.

In addition to the binding energies, the relative contributions of the fitted N 1s components before and after metal adsorption are presented in Table 5, providing a quantitative characterization of the changes in the N 1s spectral profiles (Figure S6). Metal adsorption resulted in a redistribution of the relative contributions of the fitted components, although the extent and direction of these changes depended on the sorbent and metal ion. The changes in both the N 1s binding energies and spectral profiles support the involvement of nitrogen-containing functional groups in Ni(II) and Co(II) binding.

Notably, before metal adsorption, the main N 1s component of CS/SiO_2_–APTES (399.0 eV) was very close to that of CS/SiO_2_ (398.9 eV), whereas a higher binding energy was observed for SiO_2_–APTES (399.4 eV). In addition, the two chitosan-containing materials exhibited similar changes in their N 1s spectral profiles upon Ni(II) and Co(II) adsorption, whereas a different pattern of spectral changes was observed for SiO_2_–APTES. These observations suggest a substantial contribution of the immobilized chitosan phase to the surface nitrogen environment of CS/SiO_2_–APTES and are consistent with the generally comparable adsorption behavior of the two chitosan-containing composites. Nevertheless, the possible involvement of APTES-derived nitrogen-containing groups in metal binding cannot be excluded, as their contributions cannot be reliably distinguished from those of chitosan due to overlapping N 1s signals.

## 4. Conclusions

In this work, a series of chitosan–silica composites was prepared to investigate the effects of chitosan loading and preliminary APTES functionalization of the silica surface on their physicochemical properties and Ni(II) and Co(II) adsorption behavior.

Direct precipitation in the presence of SiO_2_ resulted in a more dispersed chitosan phase compared with the large agglomerates characteristic of the pristine polymer. This observation was consistent with the higher calculated nitrogen-normalized metal uptake of the supported composites compared with pristine chitosan, suggesting that the organization of the polymer-containing phase may contribute to more effective utilization of chitosan-derived functional groups. The most pronounced changes in the textural characteristics and adsorption properties were already observed at a relatively low chitosan loading of 5 wt.%, whereas further increasing the polymer content to 10 and 20 wt.% produced comparatively smaller additional changes. Notably, chitosan modification improved Ni(II) and Co(II) uptake despite a decrease in the specific surface area and pore volume of the composites. Thus, increasing the amount of immobilized chitosan did not result in a proportional improvement in metal uptake.

Preliminary APTES functionalization was associated with a more uniform surface morphology of the resulting CS/SiO_2_–APTES composite compared with directly prepared CS/SiO_2_. Despite differences in morphology, the chitosan-containing composites exhibited relatively similar equilibrium uptake of Ni(II) and Co(II), with only modest variations observed over the investigated concentration range of 10–50 mg/L. The directly prepared CS/SiO_2_ composites also exhibited a more rapid initial metal uptake than CS/SiO_2_–APTES. FTIR and XPS analyses further revealed differences in the spectral response of the materials following Ni(II) and Co(II) adsorption. The directly prepared CS/SiO_2_ composite exhibited more pronounced shifts in the silanol-related FTIR band, whereas changes in the chitosan-associated bands were relatively minor. In contrast, CS/SiO_2_–APTES showed only minor changes in the corresponding spectral regions. Similarly, the main N 1s XPS component exhibited larger binding-energy shifts for CS/SiO_2_ than for CS/SiO_2_–APTES. Nevertheless, the N 1s spectral characteristics of CS/SiO_2_–APTES were closer to those of directly prepared CS/SiO_2_ than to SiO_2_–APTES, both before and after metal adsorption, suggesting a substantial contribution of immobilized chitosan to the surface nitrogen environment of the three-component composite. These results indicate that differences in the immobilization strategy are accompanied by variations in composite morphology, adsorption kinetics, and spectral response following metal uptake. However, equilibrium metal uptake remains relatively similar under the investigated conditions.

An additional important observation was the substantial decrease in the adsorption performance of SiO_2_–APTES after exposure to the acidic medium and subsequent neutralization, accompanied by an approximately twofold reduction in the APTES-derived organic fraction. Similar treatment during chitosan immobilization was also associated with partial loss of the APTES-derived phase. However, the deposition of chitosan increased the overall organic content of CS/SiO_2_–APTES to a level comparable to that of the original SiO_2_–APTES. Despite this similarity, the chitosan-containing composite exhibited considerably lower metal removal at an initial concentration of 10 mg/L. These findings suggest that metal-ion uptake may be influenced not only by the total organic content but also by the chemical nature and state of the surface functional groups. They also highlight the importance of preserving the functionalized surface during subsequent preparation steps in the synthesis of multicomponent polymer-inorganic materials.

From a materials-design perspective, the present results suggest that the optimization of supported chitosan sorbents should consider not only polymer loading but also the organization of the immobilized polymer phase and the chemical nature of its functional groups, which may influence their accessibility and effectiveness in metal binding. Direct precipitation onto a high-surface-area support provides a simple approach for dispersing relatively small amounts of chitosan and may serve as a basis for subsequent chemical modification of the immobilized polymer, for example, through the introduction of additional functional groups or groups with higher selectivity toward specific metal ions. At the same time, the stability of the polymer-containing phase should be considered, particularly under acidic regeneration conditions. Further modification aimed at improving the stability of immobilized chitosan may therefore be beneficial, provided that its metal-binding properties are preserved.

Support prefunctionalization, in turn, may be useful for modifying the morphology and organization of the immobilized polymer phase. At the same time, the surface chemistry of the final material may be strongly influenced by the nature and state of the immobilized polymer. This approach may be particularly useful for immobilizing functional polymers with high affinity or selectivity toward specific metal ions but insufficient affinity for the pristine inorganic surface. Furthermore, the observed differences in the spectral response of silanol-related groups following metal adsorption suggest that surface prefunctionalization followed by polymer deposition may also be of interest in the development of materials for catalytic applications, where interactions between metal species and the support surface play an important role. However, the applicability of this approach to catalytic systems requires further investigation.

## Figures and Tables

**Table 1:** Chitosan content in CS/SiO_2_ composites determined by viscometric analysis.

Composites	m(CS) in the Initial Solution, mg	m(CS) in the Solution After Precipitation, mg	m(CS) Immobilized, mg	Immobilization Degree, %	Chitosan Content, %
5% CS/SiO_2_	~50	~0	~50	~100	5
10% CS/SiO_2_	~110	~0	~110	~100	10
20% CS/SiO_2_	~250	~10	~240	~96	19

**Table 2:** BET surface area and pore structure parameters of the samples.

Sample	Specific Surface Area (S_BET_) (m^2^/g)	Total Pore Volume (cm^3^/g)	Pore Size, nm
SiO_2_	517.9	0.804	7
Chitosan	4.6	0.009	–
5% CS/SiO_2_	332.3	0.473	4–20
10% CS/SiO_2_	328.9	0.389	4–20
20% CS/SiO_2_	224.7	0.358	4–20
SiO_2_–APTES	219.9	0.373	6–10
SiO_2_–APTES-treated	370.8	0.619	7
10% CS/SiO_2_–APTES	265.9	0.353	4–20

**Table 3:** Results of organic elemental analysis of the APTES-containing samples.

Sample	Content, %	
	C	H
SiO_2_–APTES	10.5	2.4
SiO_2_–APTES-treated	5.5	1.4
CS/SiO_2_–APTES	10.4	1.8

**Table 4:** XPS parameters of Co 2p and Ni 2p spectra for Co^2+^ and Ni^2+^-loaded sorbents.

Sorbent	Binding Energy (BE), eV		
	2*p*1/2	2*p*3/2	Shake-Up
SiO_2_–Co	797.9	782.3	803.4; 786.9
Chitosan–Co	796.2	781.0	801.9; 785.5
CS/SiO_2_–Co	797.5	781.9	803.2; 787.2
SiO_2_–APTES–Co	797.6	781.9	803.1; 787.6
CS/SiO_2_–APTES–Co	796.9	781.3	802.8; 786.5
SiO_2_–Ni	874.3	856.2	880.4; 861.8
Chitosan–Ni	872.7	855.1	878.9; 860.4
CS/SiO_2_–Ni	873.3	855.7	879.9; 861.1
SiO_2_–APTES–Ni	873.5	855.8	879.5; 861.6
CS/SiO_2_–APTES–Ni	873.5	855.9	879.6; 861.6

**Table 5:** N 1s XPS data for the composite sorbents before and after Co(II) and Ni(II) adsorption.

Sorbent	N 1s Binding Energy (BE), eV		
	Before Sorption	After Co^2+^ Sorption	After Ni^2+^ Sorption
CS/SiO_2_	398.9 (78%)	399.7 (83%)	399.6 (86%)
	400.9 (22%)	401.1 (17%)	401.2 (14%)
SiO_2_–APTES	399.4 (70%)	399.8 (51%)	399.7 (78%)
	401.3 (30%)	402.0 (49%)	401.6 (22%)
CS/SiO_2_–APTES	399.0 (72%)	399.3 (91%)	399.4 (88%)
	401.3 (28%)	401.8 (9%)	401.8 (12%)

## Data Availability

The data that support the findings of this study are available from the corresponding author upon reasonable request.

## Supplementary Materials

The following supporting information can be downloaded at https://www.mdpi.com/article/10.3390/polym18212610/s1, Figure S1: FTIR spectra of SiO_2_–APTES (a), CS/SiO_2_–APTES (b), and SiO_2_–APTES–treated (c); Figure S2: TGA curves of SiO_2_, SiO_2_–APTES, SiO_2_–APTES–treated and CS/SiO_2_–APTES composites; Section S1: Calculation of N-normalized metal uptake; Table S1: N-normalized uptake of Ni(II) and Co(II) calculated using two approaches to account for the contribution of the SiO_2_ support at an initial metal concentration of 10 mg/L; Table S2: Langmuir and Freundlich isotherm parameters for Ni(II) and Co(II) adsorption onto the investigated sorbents; Table S3: Kinetic parameters of the pseudo-first-order (PFO) and pseudo-second-order (PSO) models for Ni(II) and Co(II) adsorption onto different sorbents; Figure S3. FTIR spectra of CS/SiO_2_ (a), CS/SiO_2_–APTES (b), and SiO_2_–APTES (c) before and after Ni(II) and Co(II) adsorption; Figure S4: Co 2p XPS spectra for SiO_2_ (a), chitosan (b), CS/SiO_2_ (c), SiO_2_–APTES (d), and CS/SiO_2_–APTES (e) after Co(II) adsorption; Figure S5: Ni 2p XPS spectra for SiO_2_ (a), chitosan (b), CS/SiO_2_ (c), SiO_2_–APTES (d), and CS/SiO_2_–APTES (e) after Ni(II) adsorption; Figure S6: N 1s XPS spectra for CS/SiO_2_ (a–c), CS/SiO_2_–APTES (d–f), and SiO_2_–APTES (g–i) before adsorption (a,d,g) and after Ni(II) (b,e,h) and Co(II) (c,f,i) adsorption.
