# Supplementary material for: Chitosan–Silica Composites: Effect of Polymer Loading and Immobilization Strategy on Surface Structure and Ni(II)/Co(II) Adsorption
Source: Polymers (Basel). 2026 Oct 22;18(21):2610. doi: 10.3390/polym18212610 (PMC13765568; doi:10.3390/polym18212610)
Supplement: Supplementary file 1 [file polymers-18-02610-s001.zip › polymers-4580054-supplementary.pdf]

Supplementary Information for:

Chitosan–Silica Composites: Effect of Polymer Loading and Immobilization Strategy on Surface Structure and Ni(II)/Co(II) Adsorption

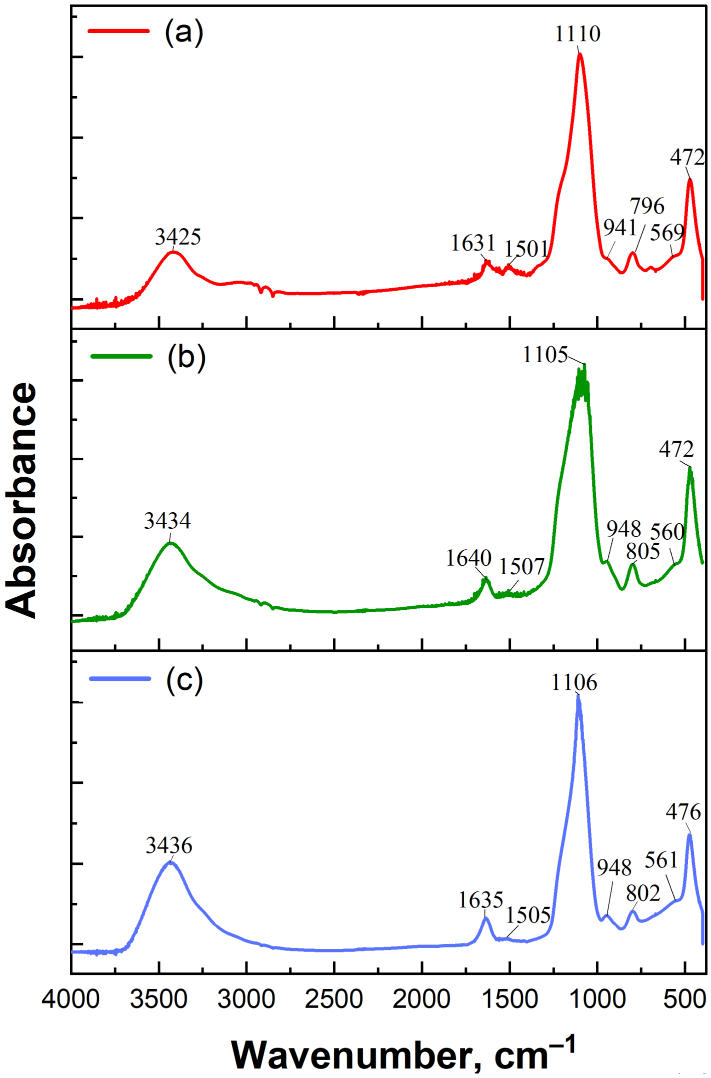

Figure S1. FTIR spectra of SiO_2–APTES (a), CS/SiO_2–APTES (b), and SiO_2–APTES–treated (c).

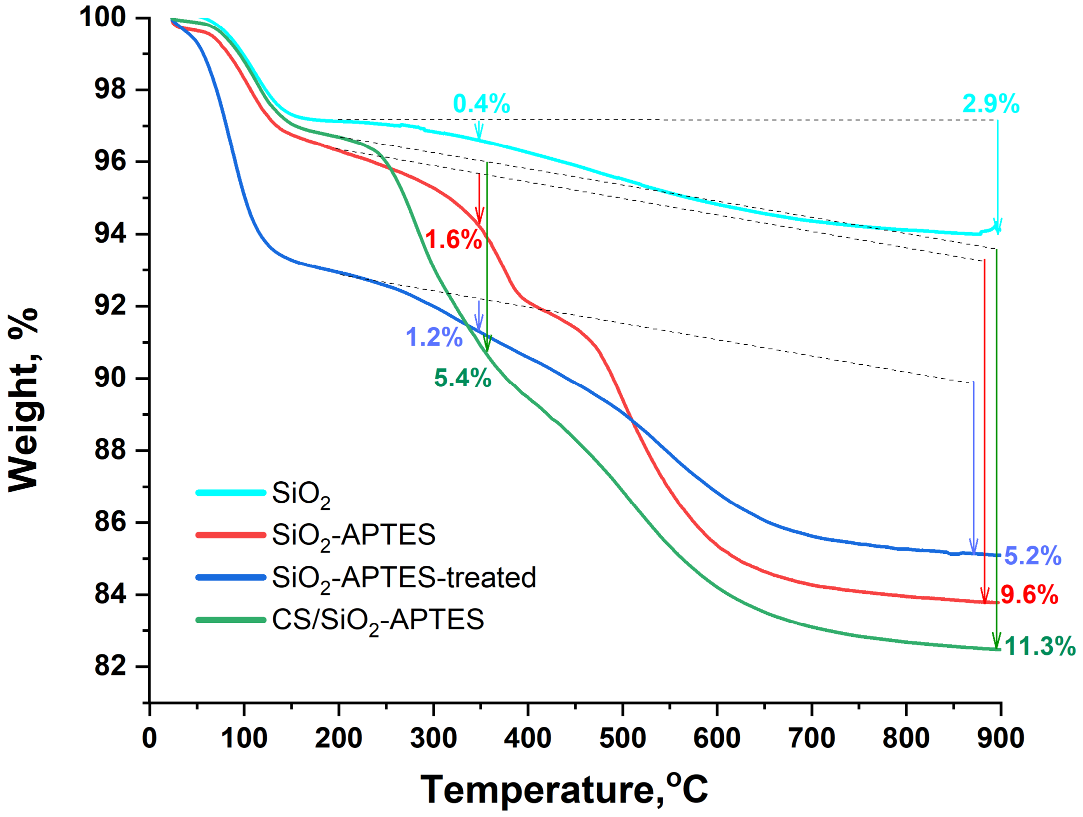

Figure S2. TGA curves of SiO_2, SiO_2–APTES, SiO_2–APTES–treated and CS/SiO_2–APTES composites.

Section S1. Calculation of N-normalized metal uptake

The nitrogen content of the samples was calculated taking into account the degree of deacetylation of chitosan (DD = 83%) and the chitosan content in the composites estimated from TGA. Based on the molar masses of the deacetylated (161.16 g/mol) and acetylated (203.19 g/mol) repeating units, the average molar mass of a chitosan repeating unit was 168.31 g/mol, corresponding to a nitrogen content of 8.32 wt.% (5.94 mmol N/g chitosan). For the TGA-estimated chitosan contents of approximately 5, 9, and 16 wt.%, the calculated nitrogen contents were 0.297, 0.535, and 0.951 mmol N/g composite, respectively.

To approximately account for the adsorption contribution of the silica support, two calculation approaches were considered. In the first approach, the experimentally measured uptake of pristine SiO_2 was directly subtracted from that of each composite:

$$q_{corr,1} = q_{comp} - q_{SiO_2} \quad \text{(S1)}$$

where $q_{corr,1}$ is the corrected metal uptake calculated using the first approach, q_{comp} is the experimentally measured metal uptake of the composite, and q_{SiO2} is the experimentally measured metal uptake of pristine SiO_2.

In the second approach, the contribution of pristine SiO_2 was scaled according to the relative BET surface area of each composite:

$$q_{corr,2} = q_{comp} - q_{SiO_2}\frac{S_{BET,comp}}{S_{BET,SiO_2}} \quad \text{(S2)}$$

where $q_{corr,2}$ is the corrected metal uptake calculated using the second approach, and $S_{BET,comp}$ and $S_{BET,SiO2}$ are the BET specific surface areas of the composite and pristine SiO_2, respectively.

The corrected amount of adsorbed metal was then normalized to the calculated amount of chitosan-derived nitrogen:

$$Q_{N,corr} = \frac{q_{corr}}{M_{metal} * n_N} \times 100 \quad \text{(S3)}$$

where $Q_{N,corr}$ is the corrected metal-to-nitrogen molar ratio expressed as mmol metal per 100 mmol N, q_{corr} represents either $q_{corr,1}$ or $q_{corr,2}$, and nN is the calculated amount of nitrogen per gram of composite.

Table S1. N-normalized uptake of Ni(II) and Co(II) calculated using two approaches to account for the contribution of the SiO_2 support at an initial metal concentration of 10 mg/L.

Sorbent	TGA-estimated CS, wt.%	N, mmol/g	Ni, mmol/100 mmol N		Co, mmol/100 mmol N	
			Full subtraction	BET-corrected	Full subtraction	BET-corrected
Pristine chitosan	100	5.942	0.9	0.9	0.6	0.6
5%CS/SiO_2	5	0.297	2.0	6.6	5.4	10.1
10%CS/SiO_2	9	0.535	1.6	4.2	3.3	5.9
20%CS/SiO_2	16	0.951	1.6	3.9	2.7	5.0

Table S2. Langmuir and Freundlich isotherm parameters for Ni(II) and Co(II) adsorption onto the investigated sorbents.

Adsorbent	Langmuir			Freundlich		
	q_{max}, mg/g	K_L, L/mg	R^2_L	1/n	K_F	R^2_F
**Ni(II)**						
SiO_2	3.318	0.379	0.975	0.171	1.702	0.990
5%CS/SiO_2	6.536	0.134	0.984	0.392	1.415	0.993
10%CS/SiO_2	8.606	0.101	0.981	0.479	1.334	0.995
CS/SiO_2–APTES	8.389	0.189	0.984	0.373	2.132	0.998
SiO_2–APTES-treated	9.398	0.059	0.997	0.555	0.927	0.986
**Co(II)**						
SiO_2	2.865	0.657	0.945	0.110	1.874	0.998
5%CS/SiO_2	6.329	0.233	0.952	0.312	1.990	0.993
10%CS/SiO_2	7.386	0.225	0.955	0.343	2.125	0.997
CS/SiO_2–APTES	8.347	0.187	0.974	0.379	2.079	0.997
SiO_2–APTES–treated	12.870	0.039	0.998	0.625	0.837	0.979

Note: The Langmuir-derived q_{max} values are model-based estimates.

Table S3. Kinetic parameters of the pseudo-first-order (PFO) and pseudo-second-order (PSO) models for Ni(II) and Co(II) adsorption onto different sorbents.

Note: Kinetic parameters were fitted using data from 0–720 min. Experimental equilibrium uptake values

**Adsorbent**	**Pseudo-first order model**			**Pseudo-second order model**			**$q_{e, exp}$ mg/g**
	**$q_{e, calc}$ mg/g**	**k_1, min^{-1}**	**R^2**	**$q_{e, calc}$ mg/g**	**k_2, $g \cdot mg^{-1} \cdot min^{-1}$**	**R^2**	
Ni(II)							
SiO_2	2.166	0.018	0.988	2.441	0.009	0.993	2.3
5%CS/SiO_2	2.485	0.122	0.965	2.608	0.079	0.990	2.5
10%CS/SiO_2	2.663	0.191	0.989	2.727	0.178	0.994	2.7
CS/SiO_2–APTES	2.933	0.021	0.957	3.297	0.008	0.985	3.3
SiO_2–APTES-treated	2.207	0.025	0.981	2.442	0.013	0.971	2.2
Co(II)							
SiO_2	2.169	0.022	0.988	2.415	0.011	0.978	2.3
5%CS/SiO_2	2.954	0.095	0.975	3.115	0.050	0.998	3.1
10%CS/SiO_2	3.145	0.170	0.978	3.259	0.104	0.992	3.3
CS/SiO_2–APTES	2.980	0.025	0.971	3.316	0.009	0.979	3.3
SiO_2–APTES–treated	2.251	0.026	0.969	2.483	0.013	0.945	2.3

were determined at 1440 min.

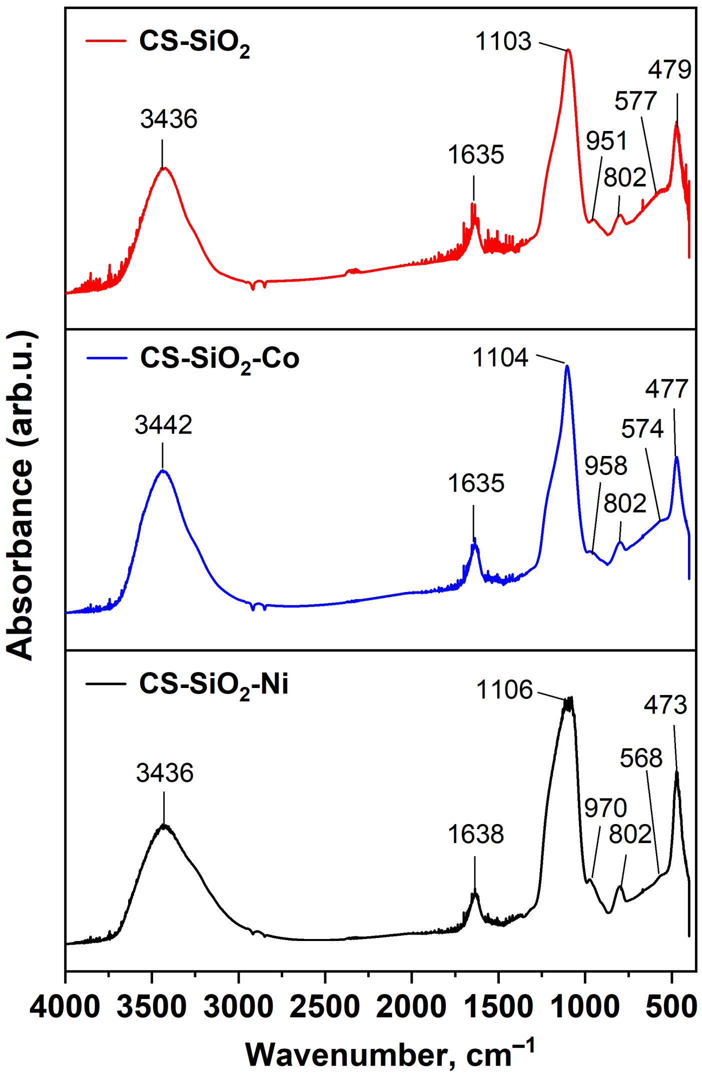
CS-SiO2
1103
479
577
3436
951
1635
802
CS-SiO2-Co
1104
477
574
3442
958
1635
802
CS-SiO2-Ni
1106
473
568
3436
970
1638
802
Absorbance (arb.u.)
4000 3500 3000 2500 2000 1500 1000 500
Wavenumber, cm−1

(a)

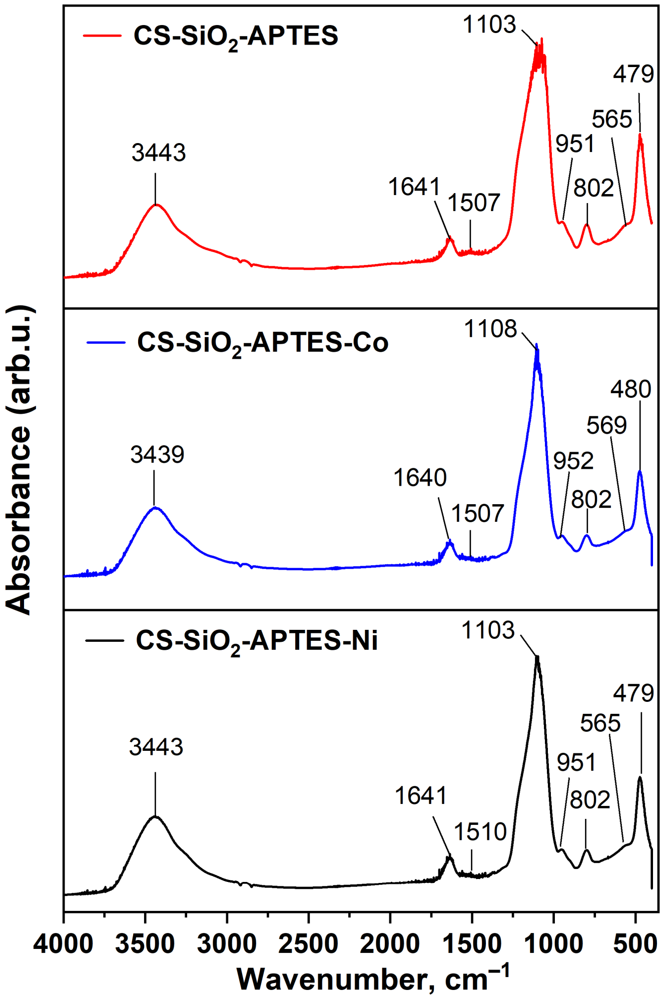

CS-SiO$_2$-APTES
CS-SiO$_2$-APTES-Co
CS-SiO$_2$-APTES-Ni
3443
1641
1507
1103
951
802
565
479
3439
1640
1507
1108
952
802
569
480
3443
1641
1510
1103
951
802
565
479
Absorbance (arb.u.)
4000
3500
3000
2500
2000
1500
1000
500
Wavenumber, cm^{-1}

(b)

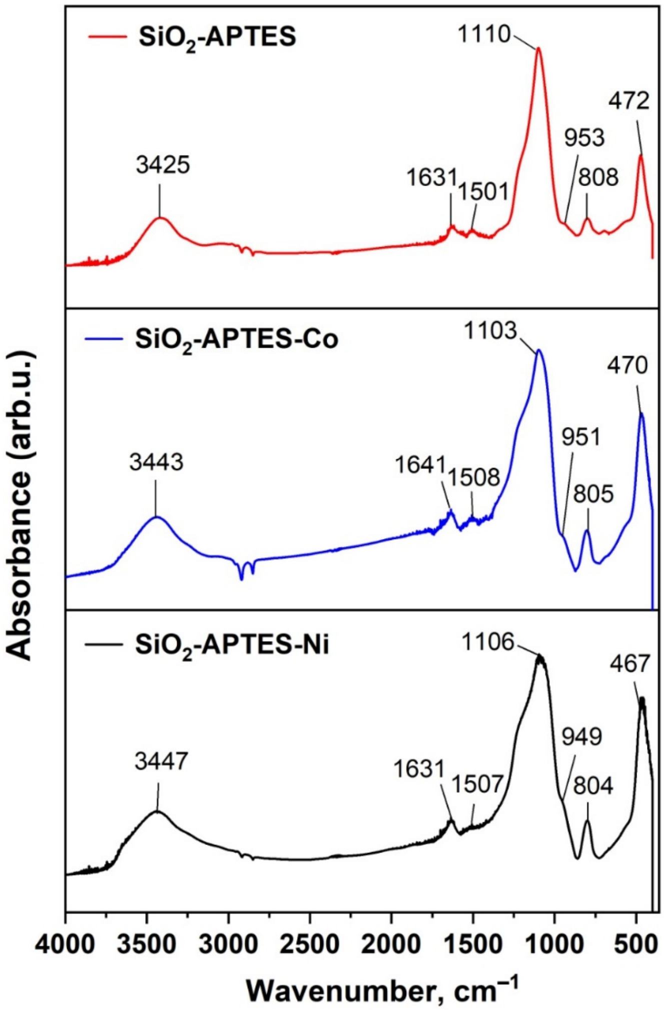

(c)

Figure S3. FTIR spectra of CS/SiO_2 (a), CS/SiO_2–APTES (b), and SiO_2–APTES (c) before and after Ni(II) and Co(II) adsorption.

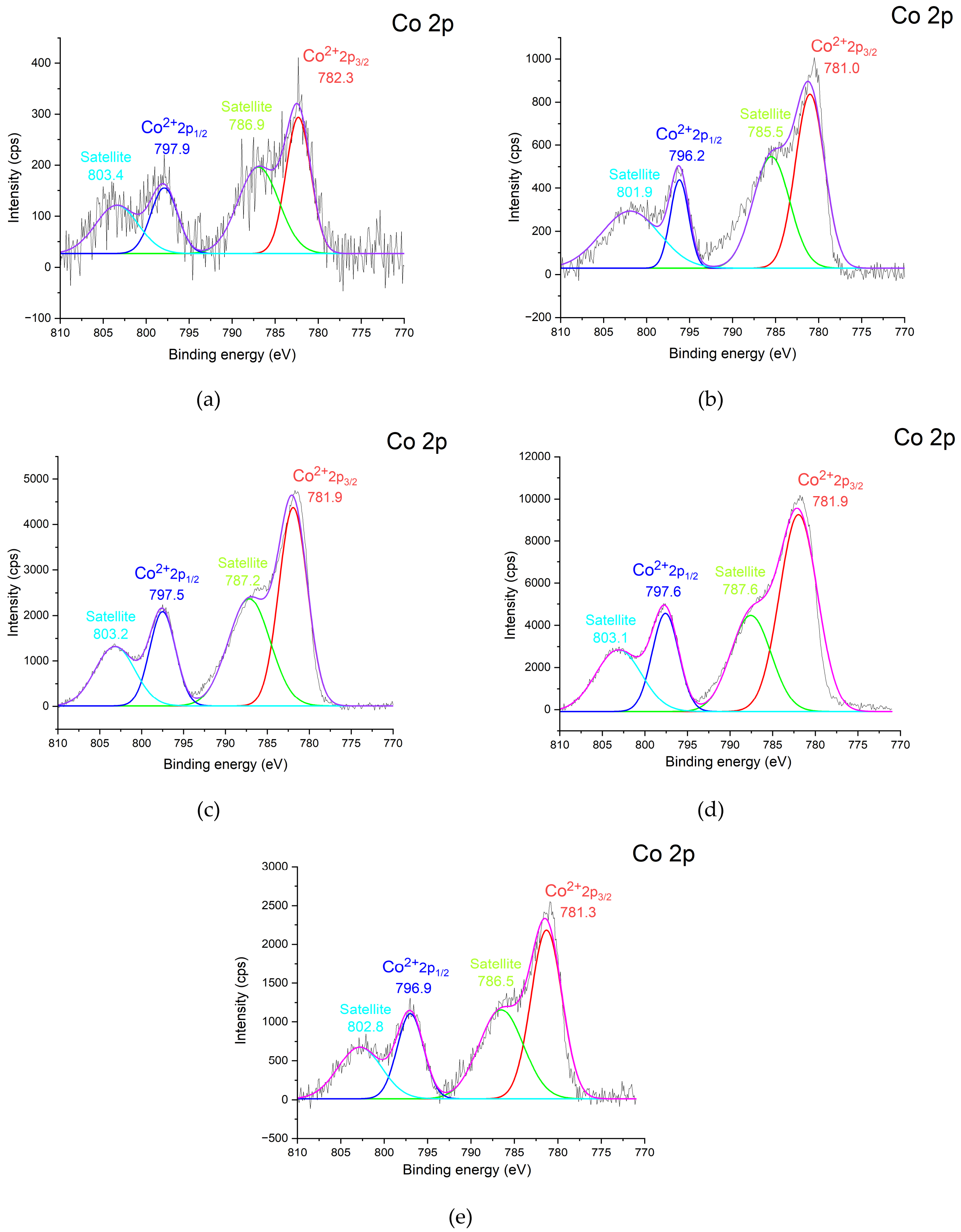

Figure S4. Co 2p XPS spectra for SiO_2 (a), chitosan (b), CS/SiO_2 (c), SiO_2–APTES (d), and CS/SiO_2–APTES (e) after Co(II) adsorption.

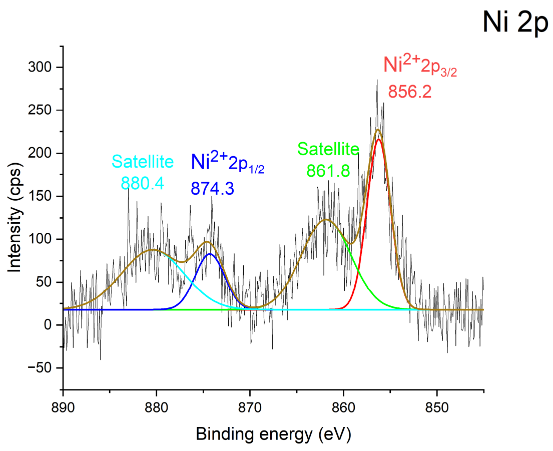

(a)

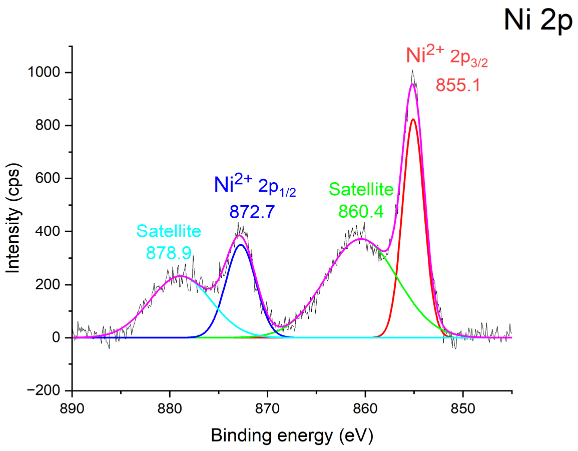

(b)

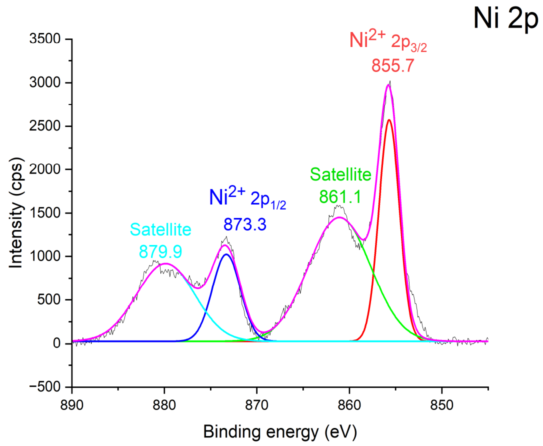

(c)

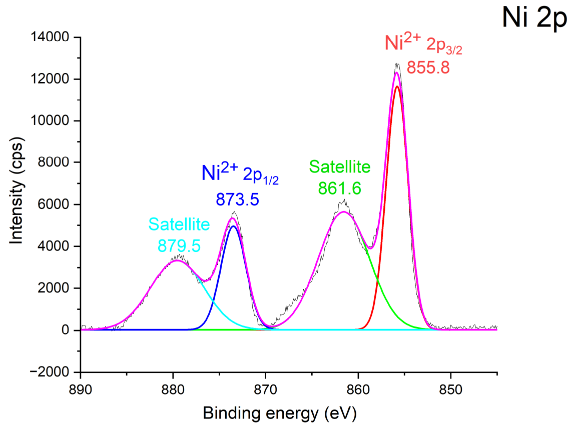

(d)

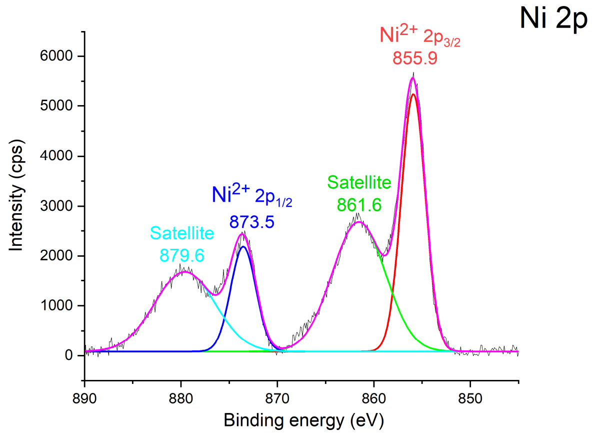

(e)

Figure S5. Ni 2p XPS spectra for SiO_2 (a), chitosan (b), CS/SiO_2 (c), SiO_2–APTES (d), and CS/SiO_2–APTES (e) after Ni(II) adsorption.

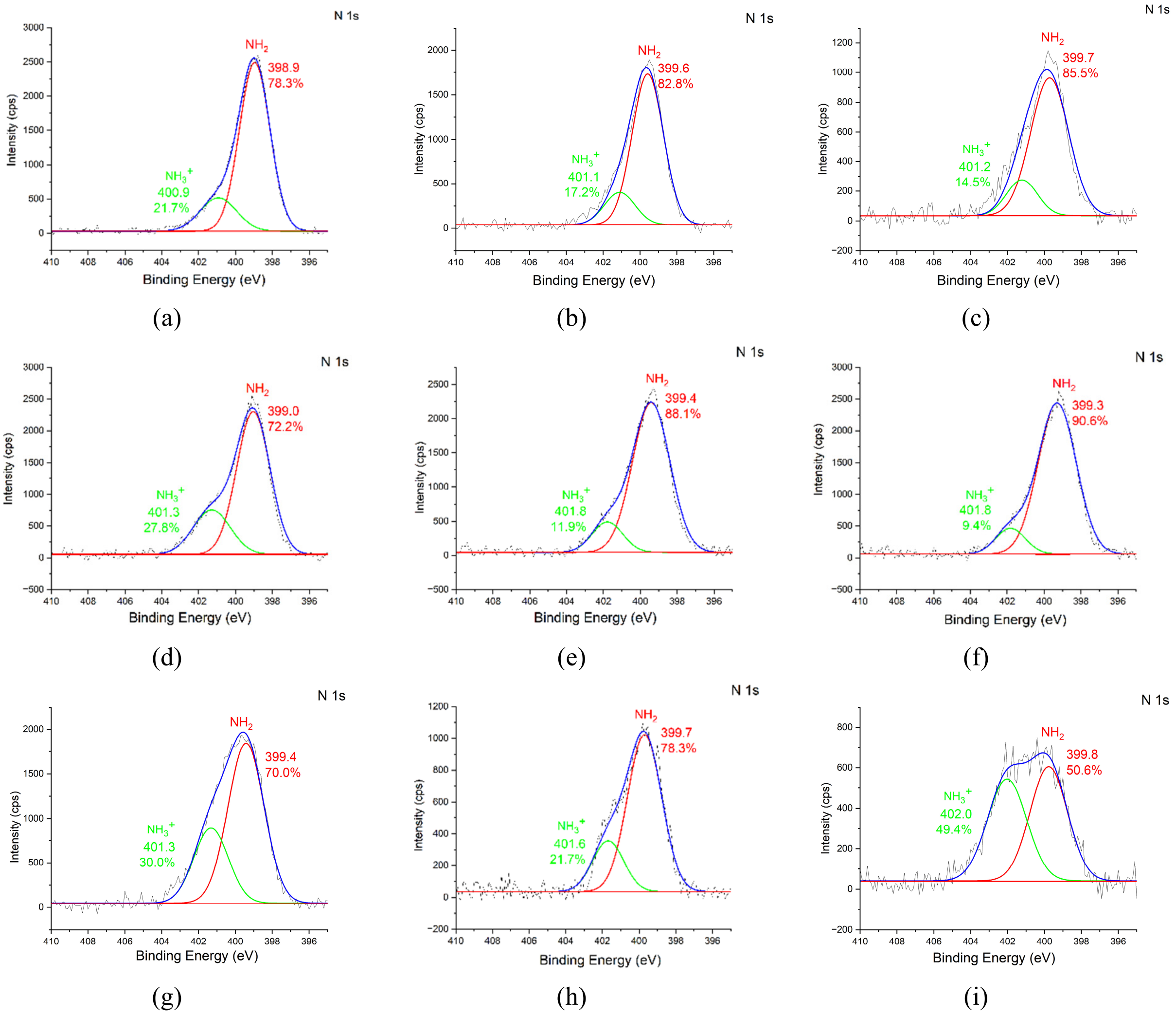

Figure S6. N 1s XPS spectra for CS/SiO_2 (a–c), CS/SiO_2–APTES (d–f), and SiO_2–APTES (g–i) before adsorption (a,d,g) and after Ni(II) (b,e,h) and Co(II) (c,f,i) adsorption.
